# Identification of key pathways and genes in polycystic ovary syndrome via integrated bioinformatics analysis and prediction of small therapeutic molecules

**DOI:** 10.1186/s12958-021-00706-3

**Published:** 2021-02-23

**Authors:** Praveenkumar Devarbhavi, Lata Telang, Basavaraj Vastrad, Anandkumar Tengli, Chanabasayya Vastrad, Iranna Kotturshetti

**Affiliations:** 1 Department of Endocrinology and Metabolism, Subbaiah Institute of Medical Sciences and Research Centre, Shimoga, Karnataka 577201 India; 2 Department of Gynaecology and Obstetrics, Subbaiah Institute of Medical Sciences and Research Centre, Shimoga, Karnataka 577201 India; 3 Department of Biochemistry, Basaveshwar College of Pharmacy, Gadag, Karnataka 582103 India; 4 Department of Pharmaceutical Chemistry, JSS College of Pharmacy, Mysuru and JSS Academy of Higher Education & Research, Mysuru, Karnataka 570015 India; 5 Biostatistics and Bioinformatics, Chanabasava Nilaya, Bharthinagar, Dharwad, Karanataka 580001 India; 6 Department of Ayurveda, Rajiv Gandhi Education Society’s Ayurvedic Medical College, Ron, Karanataka 562209 India

**Keywords:** polycystic ovary syndrome, expression profiling by high throughput sequencing, biomarkers, pathway enrichment analysis, differentially expressed gene

## Abstract

To enhance understanding of polycystic ovary syndrome (PCOS) at the molecular level; this investigation intends to examine the genes and pathways associated with PCOS by using an integrated bioinformatics analysis. Based on the expression profiling by high throughput sequencing data GSE84958 derived from the Gene Expression Omnibus (GEO) database, the differentially expressed genes (DEGs) between PCOS samples and normal controls were identified. We performed a functional enrichment analysis. A protein-protein interaction (PPI) network, miRNA- target genes and TF - target gene networks, were constructed and visualized, with which the hub gene nodes were identified. Validation of hub genes was performed by using receiver operating characteristic (ROC) and RT-PCR. Small drug molecules were predicted by using molecular docking. A total of 739 DEGs were identified, of which 360 genes were up regulated and 379 genes were down regulated. GO enrichment analysis revealed that up regulated genes were mainly involved in peptide metabolic process, organelle envelope and RNA binding and the down regulated genes were significantly enriched in plasma membrane bounded cell projection organization, neuron projection and DNA-binding transcription factor activity, RNA polymerase II-specific. REACTOME pathway enrichment analysis revealed that the up regulated genes were mainly enriched in translation and respiratory electron transport and the down regulated genes were mainly enriched in generic transcription pathway and transmembrane transport of small molecules. The top 10 hub genes (SAA1, ADCY6, POLR2K, RPS15, RPS15A, CTNND1, ESR1, NEDD4L, KNTC1 and NGFR) were identified from PPI network, miRNA - target gene network and TF - target gene network. The modules analysis showed that genes in modules were mainly associated with the transport of respiratory electrons and signaling NGF, respectively. We find a series of crucial genes along with the pathways that were most closely related with PCOS initiation and advancement. Our investigations provide a more detailed molecular mechanism for the progression of PCOS, detail information on the potential biomarkers and therapeutic targets.

## Introduction

Polycystic ovary syndrome (PCOS) is one of the most prevalent endocrine disorder around the world, with an estimated about one in 15 women worldwide [1]. PCOS exposes patients to a major psychosocial burden and is characterized by hyperandrogenism and chronic anovulation [2]. Diabetes, heart disease, obesity, non-alcoholic fatty liver disease and hypertension are the risk factors associated with PCOS [3–7]. Therefore, it is of prime importance to identify the etiological factors, molecular mechanisms, and pathways to discover novel diagnostic markers, prognostic markers and therapeutic targets for PCOS.

Numerous research strategies have recently investigated the molecular mechanisms of PCOS. High-throughput RNA sequencing technology has received extensive attention among these research strategies and has generated significant advances in the field of endocrine disorder with marked clinical applications ranging from molecular diagnosis to molecular classification, patient stratification to prognosis prediction, and discovery of new drug targets to response prediction [8]. In addition, gene expression profiling investigation on PCOS have been performed using high-throughput RNA sequencing, and several key genes and diagnostic biomarkers have been diagnosed for this syndrome, including the profiling of many of differentially expressed genes (DEGs) associated in different pathways, biological processes, or molecular functions [9]. Integrated bioinformatics analyses of expression profiling by high throughput sequencing data derived from different investigation of PCOS could help identify the novel diagnostic markers, prognostic markers and further demonstrate their related functions and potential therapeutic targets in PCOS.

Therefore, in the current investigation, the dataset (GSE84958) was then retrieved from the publicly available Gene Expression Omnibus database (GEO, http://www.ncbi.nlm.nih.gov/geo/) [10] to identify DEGs and the associated biological processes PCOS using comprehensive bioinformatics analyses. The DEGs were subjected to functional enrichment and pathway analyses; moreover, a protein-protein interaction (PPI) network, miRNAs - target gene regulatory network and TFs - target gene regulatory network were constructed to screen for key genes, miRNA and TFs. The aim of this investigation was to identify key genes and pathways in PCOS using bioinformatics analysis, and then to explore the molecular mechanisms of PCOS and categorize new potential diagnostic therapeutic biomarkers of PCOS. We anticipated that these investigations will provide further understanding of PCOS pathogenesis and advancement at the molecular level.

## Materials and Methods

### RNA sequencing data

Expression profiling by high throughput sequencing dataset GSE84958 was downloaded from NCBI-GEO, a public database of next-generation sequencing, to filter the DEGs between PCOS and normal control. The expression profiling by high throughput sequencing GSE84958 was based on GPL16791 platforms (Illumina HiSeq 2500 (Homo sapiens)) and consisted of 30 PCOS samples and 23 normal control.

### Identification of DEGs

The limma [11] in R bioconductor package was used to analyze the DEGs between PCOS samples and normal control samples in the expression profiling by high throughput sequencing data of GSE84958. The adjusted P-value and [logFC] were calculated. The Benjamini & Hochberg false discovery rate method was used as a correction factor for the adjusted P-value in limma [12]. The statistically significant DEGs were identified according to *P*<0.05, and [logFC] > 2.5 for up regulated genes and [logFC] < -1.5 for down regulated genes. All results of DEGs were downloaded in text format, hierarchical clustering analysis being conducted.

### GO and pathway enrichment of DEGs in PCOS

To reflect gene functions, GO (http://geneontology.org/) [13] has been used in three terms: biological processes (BP), cellular component (CC) and molecular function (MF). ToppGene (ToppFun) (https://toppgene.cchmc.org/enrichment.jsp) [14] is an online database offering a comprehensive collection of resources for functional annotation to recognize the biological significance behind a broad list of genes. The functional enrichment analyses of DEGs, including GO analysis and REACTOME (https://reactome.org/) [15] pathway enrichment analysis, were performed using ToppGene in the present study, using the cut-off criterion P-value<0.05 and gene enrichment count>2.

### PPI networks construction and module analysis

The Search Tool for the Retrieval of Interacting Genes/Proteins (STRING: http://string-db.org/) is online biological database and website designed to evaluate PPI information [16] Proteins associated with DEGs were selected based on information in the STRING database (PPI score >0.7), and then PPI networks were constructed using Cytoscape software (http://cytoscape.org/) [17]. In this investigation, node degree [18], betweenness centrality [19], stress centrality [20] and closeness centrality [21], these constitutes a fundamental parameters in network theory, were adopted to calculate the nodes in a network. The topological properties of hub genes were calculated using Cytoscape plugin Network Analyzer. The PEWCC1 (http://apps.cytoscape.org/apps/PEWCC1) [22], a plugin for Cytoscape, was used to screen the modules of the PPI network. The criteria were set as follows: degree cutoff=2, node score cutoff=0.2, k-core=2 and maximum depth=100. Moreover, the GO and pathway enrichment analysis were performed for DEGs in these modules.

### Construction of miRNA - target regulatory network

Furthermore, the target genes of the significant target genes were predicted by using miRNet database (https://www.mirnet.ca/) [23], when the miRNAs shared a common target gene. Finally, the miRNA - target genes regulatory network depicted interactions between miRNAs and their potential targets in PCOS were visualized by using Cytoscape.

### Construction of TF - target regulatory network

Furthermore, the target genes of the significant target genes were predicted by using TF database (https://www.mirnet.ca/) [23], when the TFs shared common target genes. Finally, the TF- target genes regulatory network depicted interactions between TFs and their potential targets in PCOS were visualized by using Cytoscape.

### Receiver operating characteristic (ROC) curve analysis

The ROC curve was used to evaluate classifiers in bioinformatics applications. To further assess the predictive accuracy of the hub genes, ROC analysis was performed to discriminate PCOS from normal control. ROC curves for hub genes were generated using pROC in R [24] based on the obtained hub genes and their expression profiling by high throughput sequencing data. The area under the ROC curve (AUC) was determined and used to compare the diagnostic value of hub genes.

### Validation of the expression levels of candidate genes by RT-PCR

Total RNA was extracted from PCOS (UWB1.289 (ATCC® CRL-2945™)) and normal ovarian cell line (MES-OV (ATCC® CRL-3272™)) using TRI Reagent® (Sigma, USA). The Reverse transcription cDNA kit (Thermo Fisher Scientific, Waltham, MA, USA) and 7 Flex real-time PCR system (Thermo Fisher Scientific, Waltham, MA, USA) were used for reverse transcription and real-time quantitative reverse transcriptase polymerase chain reaction (qRT-PCR) assay. Polymerase chain reaction primer sequences are listed in Table 1. β-actin was used as an internal control for quantification. The relative expression levels of target transcripts were calculated using the 2^-∆∆Ct^ method [25]. The thermocycling conditions used for RT-PCR were as follows: initial denaturation at 95°C for 15 min, followed by 40 cycles at 95°C for 10 sec, 60°C for 20 sec and 72°C for 20 sec.

**Table 1:** Primers used for quantitative PCR

Primer sequence (5'→3')		
Gene	Forward	Reverse
SAA1	TCGTTCCTTGGCGAGGCTTTTG	AGGTCCCCTTTTGGCAGCATCA
ADCY6	CTCCTGGTCCCTAAAGTGGAT	GGAGGCAGCTCATATAGCGG
POLR2K	GGAGAGTGTCACACAGAAAATGA	TCGAGCATCAAAAACGACCAAT
RPS15	CCCGAGATGATCGGCCACTA	CCATGCTTTACGGGCTTGTAG
RPS15A	CTCCAAAGTCATCGTCCGGTT	TGAGTTGCACGTCAAATCTGG
CTNND1	GTGACAACACGGACAGTACAG	TTCTTGCGGAAATCACGACCC
ESR1	CCCACTCAACAGCGTGTCTC	CGTCGATTATCTGAATTTGGCCT
NEDD4L	GACATGGAGCATGGATGGGAA	GTTCGGCCTAAATTGTCCACT
KNTC1	ACCTGAGTGTCGGTTCAAGAA	CACTGATTGGTCGGCTACAATAA
NGFR	CCTACGGCTACTACCAGGATG	CACACGGTGTTCTGCTTGT

### Molecular docking studies

Surflex-docking studies of the standard drug molecule used in polycystic ovary syndrome were used on over expressed genes and were collected from PDB data bank using perpetual SYBYL-X 2.0 software. Using ChemDraw Software, all the drug molecules were illustrated, imported and saved in sdf. templet using open babel free software. The protein structures of POLR2K (), RPS15, RPS15 alpha and SAA1 of their co-crystallised protein of PDB code 1LE9, 3OW2, 1G1X and 4IP8 respectively were extracted from Protein Data Bank [26–28]. Gasteiger Huckel (GH) charges were applied along with the TRIPOS force field to all the drug molecules and is standard for the structure optimization process. In addition, energy minimization was achieved using MMFF94s and MMFF94 algorithm methods. The protein preparation was carried out after incorporation of protein. The co-crystallized ligand was extracted from the crystal structure and all water molecules; more hydrogen was added and the side chain was set. For energy minimisation, the TRIPOS force field was used. The interaction efficiency of the compounds with the receptor was represented in kcal / mol units by the Surflex-Dock score. The interaction between the protein and the ligand, the best pose was incorporated into the molecular area. The visualization of ligand interaction with receptor is done by using discovery studio visualizer.

## Results

### Identification of DEGs

**Fig. 1:**
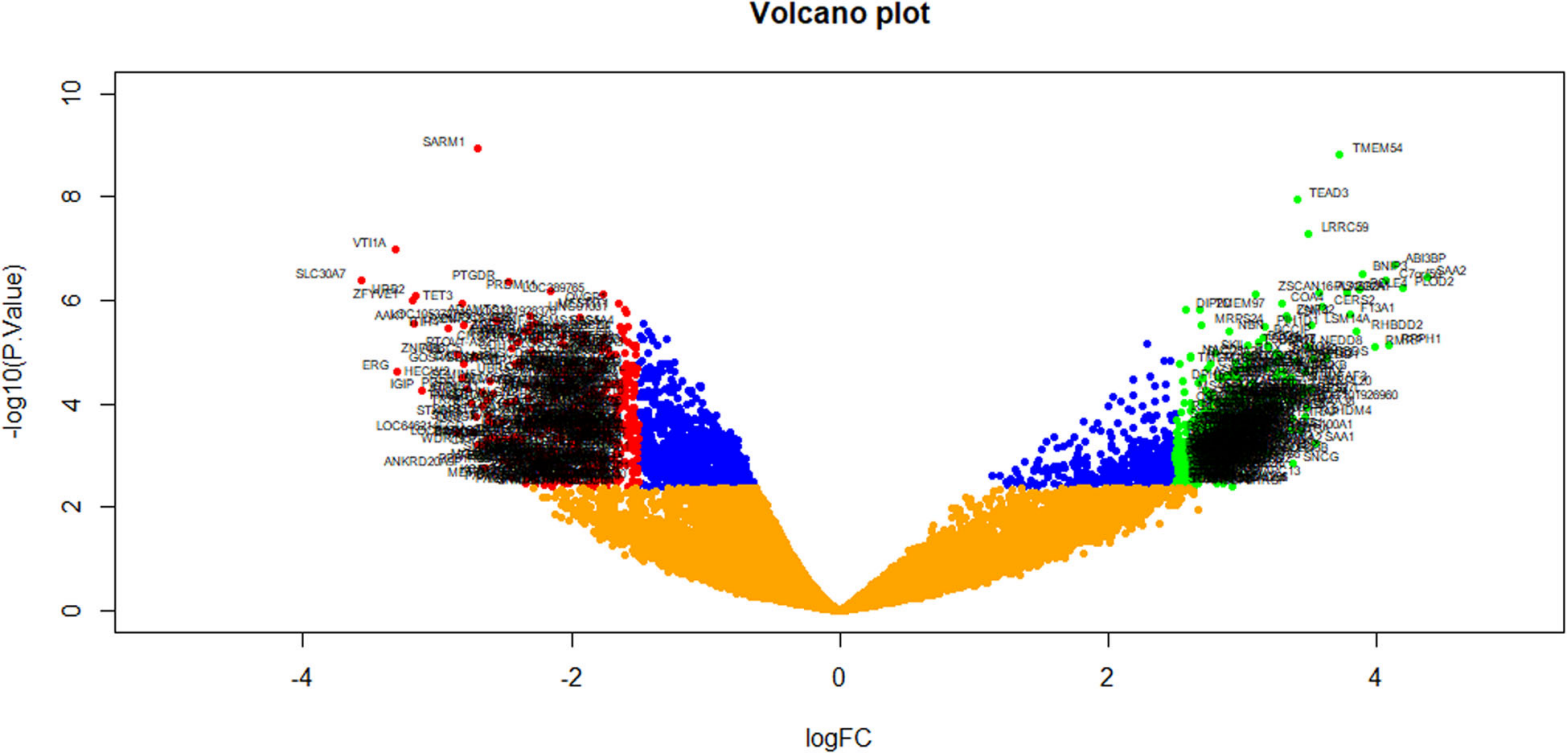
Volcano plot of differentially expressed genes. Genes with a significant change of more than two-fold were selected. Green dot represented up regulated significant genes and red dot represented down regulated significant genes

**Fig. 2:**
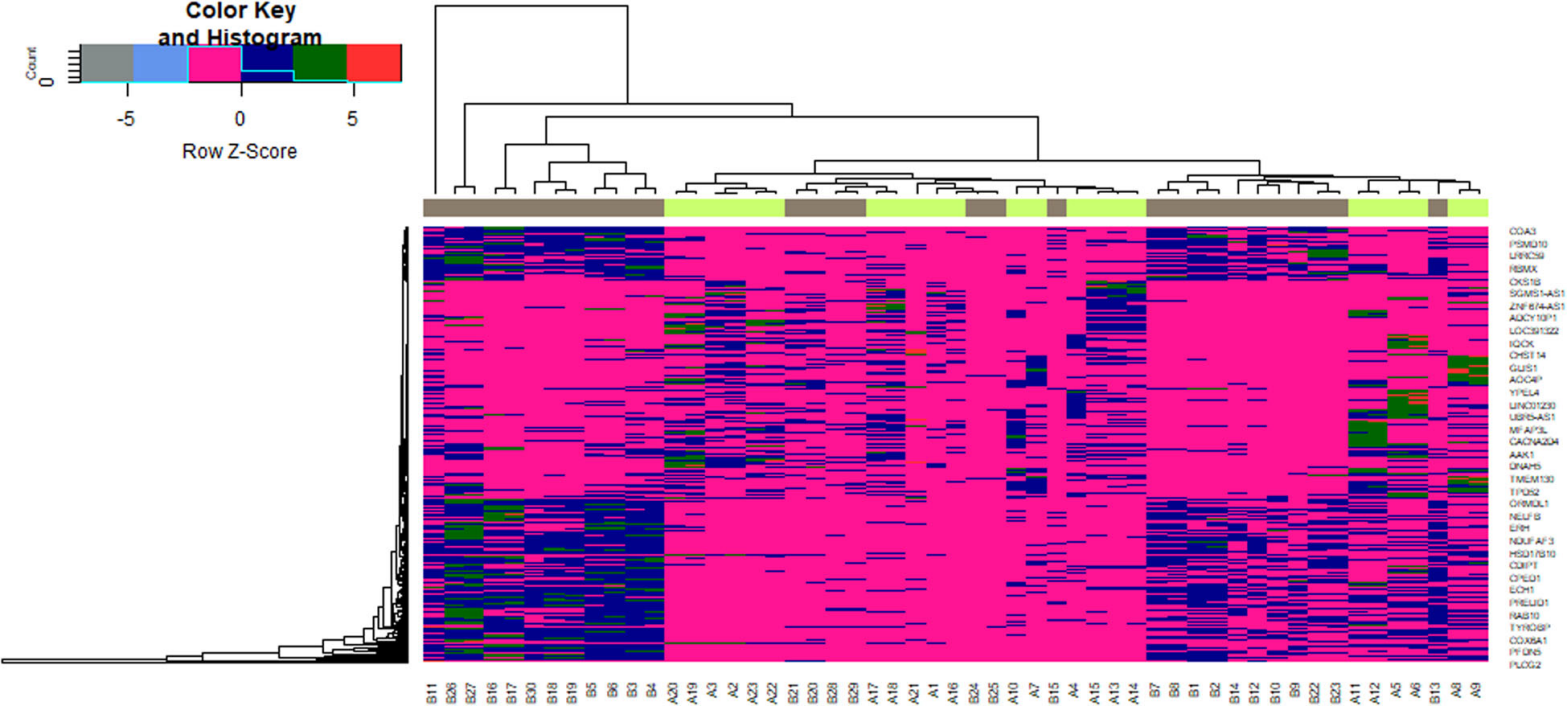
Heat map of differentially expressed genes. Legend on the top left indicate log fold change of genes. (A1 – A2 = normal control samples; B1 – B30 = PCOS samples)

Expression profiling by high throughput sequencing dataset was obtained from the National Center for Biotechnology Information GEO database containing PCOS samples and normal control samples: GSE84958. Then, the R package named “limma” was processed for analysis with adjusted *P* < 0.05, and [logFC] > 2.5 for up regulated genes and [logFC] < -1.5 for down regulated genes. All DEGs were displayed in volcano maps (Fig. 1). A total of 739 DEGs including 360 up regulated and 379 down regulated genes (Table 2) were identified in PCOS samples compared to normal control samples. The results are shown in the heatmap (Fig. 2).

**Table 2:** The statistical metrics for key differentially expressed genes (DEGs)

Gene Symbol	logFC	***p*** Value	adj. P.Val	t value	Regulation	Gene Name
SAA2	4.381364	3.5E-07	0.00095	5.718643	Up	serum amyloid A2
PLOD2	4.201209	5.64E-07	0.001025	5.593469	Up	procollagen-lysine,2-oxoglutarate 5-dioxygenase 2
ABI3BP	4.13442	2.07E-07	0.000779	5.856015	Up	ABI family member 3 binding protein
RPPH1	4.09338	7.43E-06	0.002243	4.901502	Up	ribonuclease P RNA component H1
C7orf50	4.074966	4.01E-07	0.00095	5.683289	Up	chromosome 7 open reading frame 50
RMRP	3.989952	7.74E-06	0.002243	4.890252	Up	RNA component of mitochondrial RNA processing endoribonuclease
BNIP3	3.899781	2.99E-07	0.00095	5.760151	Up	BCL2 interacting protein 3
POLE4	3.874065	6.35E-07	0.001025	5.562056	Up	DNA polymerase epsilon 4, accessory subunit
RHBDD2	3.854997	4.02E-06	0.001884	5.068914	Up	rhomboid domain containing 2
F13A1	3.806046	1.82E-06	0.001545	5.283097	Up	coagulation factor XIII A chain
SIVA1	3.784719	7.02E-07	0.001025	5.535746	Up	SIVA1 apoptosis inducing factor
TMEM54	3.721	1.5E-09	1.98E-05	7.116347	Up	transmembrane protein 54
SBDS	3.630263	1.28E-05	0.002885	4.75139	Up	SBDS ribosome maturation factor
CERS2	3.600241	1.32E-06	0.001336	5.368773	Up	ceramide synthase 2
PLA2G2A	3.575964	6.92E-07	0.001025	5.539611	Up	phospholipase A2 group IIA
SAA1	3.551756	0.000565	0.017754	3.640098	Up	serum amyloid A1
PHB2	3.526406	1.39E-05	0.003049	4.728928	Up	prohibitin 2
LSM14A	3.514098	2.94E-06	0.001776	5.153832	Up	LSM14A mRNA processing body assembly factor
MRPL20	3.513535	4.93E-05	0.005042	4.370518	Up	mitochondrial ribosomal protein L20
NEDD8	3.493441	7.9E-06	0.002265	4.884608	Up	NEDD8 ubiquitin like modifier
LRRC59	3.487927	5.07E-08	0.000334	6.219575	Up	leucine rich repeat containing 59
NAA38	3.483141	0.000119	0.008042	4.113375	Up	N (alpha)-acetyltransferase 38, NatC auxiliary subunit
R3HDM4	3.472879	0.000181	0.009987	3.989599	Up	R3H domain containing 4
PI4KB	3.463829	2.36E-05	0.003497	4.579687	Up	phosphatidylinositol 4-kinase beta
RPS27L	3.462436	6.2E-05	0.005556	4.304481	Up	ribosomal protein S27 like
UBL5	3.458582	4.83E-05	0.005015	4.376605	Up	ubiquitin like 5
S100A1	3.431536	0.000323	0.013388	3.813673	Up	S100 calcium binding protein A1
TEAD3	3.414631	1.1E-08	9.64E-05	6.611182	Up	TEA domain transcription factor 3
PRELID1	3.395554	1.51E-05	0.003137	4.706077	Up	PRELI domain containing 1
SNCG	3.381526	0.001426	0.0285	3.342577	Up	synuclein gamma
NDUFAF3	3.380114	3.84E-05	0.00453	4.442043	Up	NADH:ubiquinoneoxidoreductase complex assembly factor 3
LOC101926960	3.379084	8.99E-05	0.006783	4.196554	Up	uncharacterized LOC101926960
MRAP	3.376232	0.000186	0.010117	3.981504	Up	melanocortin 2 receptor accessory protein
EIF1AX	3.363678	3.15E-05	0.004025	4.498903	Up	eukaryotic translation initiation factor 1A X-linked
FIS1	3.36153	0.000316	0.01319	3.819875	Up	fission, mitochondrial 1
LSM4	3.342924	2.22E-06	0.001669	5.229869	Up	LSM4 homolog, U6 small nuclear RNA and mRNA degradation associated
MRPL24	3.341155	8.03E-05	0.006322	4.229412	Up	mitochondrial ribosomal protein L24
ZNF32	3.328313	2.03E-06	0.001623	5.253128	Up	zinc finger protein 32
SAP18	3.320223	3.41E-05	0.004196	4.47629	Up	Sin3A associated protein 18
ZFAND6	3.310728	6.93E-05	0.005946	4.272145	Up	zinc finger AN1-type containing 6
PLCG2	3.309309	2.45E-05	0.003542	4.570246	Up	phospholipase C gamma 2
COA4	3.297112	1.12E-06	0.001179	5.412446	Up	cytochrome c oxidase assembly factor 4 homolog
GTF3A	3.27873	0.000407	0.015016	3.742231	Up	general transcription factor IIIA
SMIM19	3.278578	1.23E-05	0.002829	4.763305	Up	small integral membrane protein 19
DYNLL2	3.274028	1.21E-05	0.002818	4.76751	Up	dynein light chain LC8-type 2
WSB2	3.272902	0.000138	0.008726	4.070129	Up	WD repeat and SOCS box containing 2
MMAB	3.272634	2.33E-05	0.003497	4.583501	Up	metabolism of cobalamin associated B
COX5B	3.250497	8.68E-05	0.006676	4.206576	Up	cytochrome c oxidase subunit 5B
ZNF706	3.247455	1.57E-05	0.003137	4.695098	Up	zinc finger protein 706
SUMO3	3.244703	1.07E-05	0.002689	4.799887	Up	small ubiquitin like modifier 3
ZHX1	3.244233	2.98E-05	0.003952	4.514129	Up	zinc fingers and homeoboxes 1
DCAF6	3.218358	7.16E-05	0.005962	4.263031	Up	DDB1 and CUL4 associated factor 6
NDUFA2	3.202606	0.00011	0.007611	4.136503	Up	NADH:ubiquinoneoxidoreductase subunit A2
COMT	3.194553	7.49E-06	0.002243	4.899155	Up	catechol-O-methyltransferase
USP24	3.193685	7.7E-06	0.002243	4.891689	Up	ubiquitin specific peptidase 24
CLEC3B	3.192924	0.000932	0.023147	3.480824	Up	C-type lectin domain family 3 member B
S100A16	3.181908	2.08E-05	0.00341	4.615343	Up	S100 calcium binding protein A16
PIH1D1	3.168542	3.25E-06	0.001776	5.126645	Up	PIH1 domain containing 1
PEX2	3.15767	6.91E-05	0.005946	4.273176	Up	peroxisomal biogenesis factor 2
BCCIP	3.156384	5.08E-06	0.002061	5.005401	Up	BRCA2 and CDKN1A interacting protein
UCHL1	3.144433	0.000603	0.018411	3.61943	Up	ubiquitin C-terminal hydrolase L1
CCND2	3.137527	0.000795	0.021152	3.531905	Up	cyclin D2
MTDH	3.134009	8.58E-05	0.006622	4.210031	Up	metadherin
ATP6V1D	3.1239	0.0001	0.007145	4.164159	Up	ATPase H+ transporting V1 subunit D
BOD1	3.11664	6.28E-06	0.002168	4.947675	Up	biorientation of chromosomes in cell division 1
MRPL12	3.114756	7.59E-05	0.006143	4.245866	Up	mitochondrial ribosomal protein L12
FOXN3	3.113552	0.000114	0.007817	4.126313	Up	forkhead box N3
POLR3GL	3.098647	0.000131	0.008485	4.084894	Up	RNA polymerase III subunit G like
ALKBH7	3.098442	0.000306	0.012934	3.830063	Up	alkB homolog 7
CDV3	3.096327	0.000108	0.007553	4.141317	Up	CDV3 homolog
ZSCAN16-AS1	3.095847	7.34E-07	0.001025	5.523754	Up	ZSCAN16 antisense RNA 1
S100A13	3.091633	5.32E-05	0.005263	4.348914	Up	S100 calcium binding protein A13
GFPT1	3.085405	5.33E-05	0.005263	4.348282	Up	glutamine--fructose-6-phosphate transaminase 1
CCSER2	3.079066	3.98E-05	0.004552	4.431771	Up	coiled-coil serine rich protein 2
PET100	3.05957	0.000152	0.009259	4.041067	Up	PET100 cytochrome c oxidase chaperone
POLR2J	3.05183	0.000358	0.014077	3.781554	Up	RNA polymerase II subunit J
SF3B6	3.047526	0.000179	0.009929	3.992753	Up	splicing factor 3b subunit 6
TSPAN17	3.044512	7.59E-06	0.002243	4.895829	Up	tetraspanin 17
ECSIT	3.043372	1.61E-05	0.003137	4.688241	Up	ECSIT signaling integrator
TMED4	3.041199	2.05E-05	0.00341	4.620554	Up	transmembrane p24 trafficking protein 4
ROMO1	3.039801	0.000339	0.013645	3.798806	Up	reactive oxygen species modulator 1
SCARNA2	3.037947	0.000602	0.018408	3.619837	Up	small Cajal body-specific RNA 2
RDX	3.036218	1.11E-05	0.002709	4.790401	Up	radixin
ATP6AP2	3.032219	0.000537	0.01724	3.656205	Up	ATPase H+ transporting accessory protein 2
MRPS6	3.031793	0.001246	0.026775	3.386766	Up	mitochondrial ribosomal protein S6
MAD2L1BP	3.030044	1.83E-05	0.003308	4.651088	Up	MAD2L1 binding protein
NNAT	3.01612	0.001284	0.027046	3.376953	Up	neuronatin
SNX8	3.011952	2.11E-05	0.00341	4.611584	Up	sorting nexin 8
ITGAV	3.006812	0.000168	0.009643	4.011701	Up	integrin subunit alpha V
TBCA	3.005083	8.86E-05	0.006772	4.200708	Up	tubulin folding cofactor A
SNAPIN	3.00314	6.24E-05	0.005556	4.30272	Up	SNAP associated protein
TIMM8B	2.999367	0.000105	0.007409	4.149862	Up	translocase of inner mitochondrial membrane 8 homolog B
TADA3	2.994876	4.72E-05	0.005	4.38301	Up	transcriptional adaptor 3
HLA-DPB1	2.992346	0.000391	0.014705	3.754948	Up	major histocompatibility complex, class II, DP beta 1
MGP	2.989607	0.000677	0.019612	3.583067	Up	matrix Gla protein
LAMTOR4	2.985466	0.000916	0.022949	3.486633	Up	late endosomal/lysosomal adaptor, MAPK and MTOR activator 4
BTF3L4	2.981702	8.91E-05	0.006776	4.199218	Up	basic transcription factor 3 like 4
TMX2	2.979038	0.000153	0.009259	4.040171	Up	thioredoxin related transmembrane protein 2
CFL2	2.977258	3.16E-05	0.004025	4.497382	Up	cofilin 2
FAM149A	2.975549	6.83E-05	0.005946	4.276377	Up	family with sequence similarity 149 member A
DTYMK	2.966972	0.000289	0.012546	3.847791	Up	deoxythymidylate kinase
MT1X	2.96256	0.000442	0.015758	3.716594	Up	metallothionein 1X
TSG101	2.95913	0.00027	0.012156	3.868591	Up	tumor susceptibility 101
CPED1	2.957683	0.000497	0.016679	3.680123	Up	cadherin like and PC-esterase domain containing 1
EXOC5	2.949442	0.000271	0.012156	3.867668	Up	exocyst complex component 5
BCYRN1	2.947993	1.57E-05	0.003137	4.695075	Up	brain cytoplasmic RNA 1
ANXA4	2.947244	2.18E-05	0.00341	4.602361	Up	annexin A4
DPP7	2.946214	8.92E-05	0.006776	4.198665	Up	dipeptidyl peptidase 7
SYVN1	2.942727	6.24E-05	0.005556	4.302587	Up	synoviolin 1
NDUFA13	2.941729	0.000735	0.020483	3.556961	Up	NADH:ubiquinoneoxidoreductase subunit A13
C9orf16	2.940481	0.000406	0.014994	3.743119	Up	chromosome 9 open reading frame 16
AMDHD2	2.940188	2.53E-05	0.00358	4.5612	Up	amidohydrolase domain containing 2
KLHL12	2.939522	2.85E-05	0.003867	4.527463	Up	kelch like family member 12
EIF2D	2.938722	0.000261	0.011982	3.878331	Up	eukaryotic translation initiation factor 2D
NDUFB2	2.935453	7.75E-05	0.006191	4.239855	Up	NADH:ubiquinoneoxidoreductase subunit B2
GLRX3	2.93158	0.001019	0.024195	3.452028	Up	glutaredoxin 3
THRSP	2.927803	0.00402	0.04933	2.990471	Up	thyroid hormone responsive
SLC25A11	2.925157	0.000548	0.017445	3.649379	Up	solute carrier family 25 member 11
NDFIP1	2.924945	8E-05	0.006317	4.230473	Up	Nedd4 family interacting protein 1
UBAC1	2.919604	0.000143	0.008978	4.058832	Up	UBA domain containing 1
SETD3	2.915915	0.000233	0.011326	3.913355	Up	SET domain containing 3, actin histidinemethyltransferase
UQCRH	2.914264	0.000206	0.010586	3.950821	Up	ubiquinol-cytochrome c reductase hinge protein
LMBRD1	2.91352	0.000153	0.009259	4.039227	Up	LMBR1 domain containing 1
C1S	2.913138	9.66E-05	0.007077	4.175345	Up	complement C1s
YIPF6	2.909794	0.000398	0.014783	3.74923	Up	Yip1 domain family member 6
COX4I1	2.908785	0.000397	0.014772	3.749898	Up	cytochrome c oxidase subunit 4I1
NBN	2.90833	3.91E-06	0.001884	5.076794	Up	nibrin
TAF7	2.908109	0.000345	0.013777	3.793503	Up	TATA-box binding protein associated factor 7
ANAPC13	2.904023	0.002551	0.038877	3.147704	Up	anaphase promoting complex subunit 13
LYVE1	2.902347	0.001203	0.026382	3.398322	Up	lymphatic vessel endothelial hyaluronan receptor 1
PSMD14	2.901936	0.000247	0.011699	3.89503	Up	proteasome 26S subunit, non-ATPase 14
FAM32A	2.900891	0.000535	0.01724	3.657406	Up	family with sequence similarity 32 member A
UROS	2.900315	0.00024	0.011528	3.904656	Up	uroporphyrinogen III synthase
OST4	2.898201	0.002022	0.034115	3.226353	Up	oligosaccharyltransferase complex subunit 4, non-catalytic
RBBP7	2.897646	0.000946	0.023286	3.476066	Up	RB binding protein 7, chromatin remodeling factor
PRG4	2.897614	4.83E-05	0.005015	4.376505	Up	proteoglycan 4
COX7A2	2.896609	0.000818	0.021597	3.522952	Up	cytochrome c oxidase subunit 7A2
HMGCL	2.895335	0.000179	0.009929	3.993321	Up	3-hydroxy-3-methylglutaryl-CoA lyase
FAM3A	2.894614	8.47E-05	0.006573	4.213715	Up	family with sequence similarity 3 member A
BAK1	2.892595	2.69E-05	0.00371	4.543614	Up	BCL2 antagonist/killer 1
ELOVL6	2.8913	7.93E-05	0.006283	4.232941	Up	ELOVL fatty acid elongase 6
CHCHD2	2.888344	0.000193	0.010317	3.970255	Up	coiled-coil-helix-coiled-coil-helix domain containing 2
PRMT1	2.886326	0.000236	0.01142	3.909722	Up	protein arginine methyltransferase 1
HCFC1R1	2.88509	0.000187	0.010126	3.979988	Up	host cell factor C1 regulator 1
RPS8	2.88263	0.000763	0.02086	3.545152	Up	ribosomal protein S8
JMJD8	2.880095	0.000466	0.016117	3.700249	Up	jumonji domain containing 8
VPS28	2.868412	0.00038	0.014539	3.763185	Up	VPS28 subunit of ESCRT-I
EIF5	2.867934	0.00037	0.014306	3.772157	Up	eukaryotic translation initiation factor 5
ADCY6	2.866913	0.000145	0.009003	4.055897	Up	adenylatecyclase 6
NDUFC2	2.865496	0.001531	0.029477	3.319099	Up	NADH:ubiquinoneoxidoreductase subunit C2
PIGS	2.862467	0.002273	0.036573	3.186856	Up	phosphatidylinositol glycan anchor biosynthesis class S
C1QA	2.861687	0.000816	0.021594	3.523683	Up	complement C1q A chain
FDX1	2.861442	0.000394	0.014749	3.752127	Up	ferredoxin 1
RBX1	2.85968	0.000974	0.023505	3.466607	Up	ring-box 1
TRIB1	2.8594	3.17E-05	0.004025	4.496868	Up	tribblespseudokinase 1
COX6B1	2.858458	0.000469	0.01616	3.698433	Up	cytochrome c oxidase subunit 6B1
MDFI	2.857869	0.000277	0.012363	3.860877	Up	MyoD family inhibitor
RASD1	2.855883	0.00079	0.021079	3.533796	Up	ras related dexamethasone induced 1
SLC40A1	2.854864	0.003422	0.045502	3.046747	Up	solute carrier family 40 member 1
POLR2K	2.854222	0.001838	0.032468	3.258347	Up	RNA polymerase II subunit K
CYB5B	2.852742	9.79E-05	0.00709	4.171566	Up	cytochrome b5 type B
C1orf115	2.850887	0.001855	0.03266	3.255182	Up	chromosome 1 open reading frame 115
EIF3L	2.846772	0.000651	0.019135	3.595545	Up	eukaryotic translation initiation factor 3 subunit L
TMEM219	2.834679	0.001098	0.025262	3.427919	Up	transmembrane protein 219
UQCR11	2.833847	0.000542	0.017312	3.652923	Up	ubiquinol-cytochrome c reductase, complex III subunit XI
AGFG1	2.83177	8.83E-05	0.006771	4.201612	Up	ArfGAP with FG repeats 1
MRPS15	2.831483	0.000629	0.018848	3.606346	Up	mitochondrial ribosomal protein S15
UBE2E1	2.82743	0.00062	0.018805	3.610605	Up	ubiquitin conjugating enzyme E2 E1
NQO1	2.827398	3.12E-05	0.004025	4.50132	Up	NAD(P) H quinone dehydrogenase 1
MORF4L1	2.825223	0.000145	0.009003	4.055546	Up	mortality factor 4 like 1
TM7SF3	2.823384	0.0001	0.007145	4.165181	Up	transmembrane 7 superfamily member 3
RPL35A	2.822157	0.000266	0.012089	3.87247	Up	ribosomal protein L35a
TMEM160	2.812975	0.000317	0.01319	3.819678	Up	transmembrane protein 160
LSM3	2.809963	0.000282	0.012404	3.854606	Up	LSM3 homolog, U6 small nuclear RNA and mRNA degradation associated
PHB	2.808084	0.000258	0.011974	3.881731	Up	prohibitin
MRPS21	2.805911	0.000295	0.012696	3.841189	Up	mitochondrial ribosomal protein S21
TMEM256	2.804853	0.003453	0.045785	3.04363	Up	transmembrane protein 256
MRPS12	2.804789	0.000223	0.011092	3.925752	Up	mitochondrial ribosomal protein S12
PLTP	2.803513	0.000258	0.011965	3.882486	Up	phospholipid transfer protein
TNPO2	2.794461	0.000852	0.022167	3.509567	Up	transportin 2
SKIL	2.793991	1.01E-05	0.002625	4.815964	Up	SKI like proto-oncogene
SEC11A	2.784473	0.001174	0.026198	3.406284	Up	SEC11 homolog A, signal peptidase complex subunit
RPS10	2.783754	0.000749	0.020638	3.550811	Up	ribosomal protein S10
APOD	2.777795	0.00264	0.039615	3.136112	Up	apolipoprotein D
RAB4A	2.777311	0.000182	0.01001	3.987809	Up	RAB4A, member RAS oncogene family
RBMX	2.774453	6.17E-05	0.005556	4.306198	Up	RNA binding motif protein X-linked
ARFIP2	2.772975	0.000459	0.016062	3.705244	Up	ADP ribosylation factor interacting protein 2
CPE	2.770836	0.001392	0.028193	3.350337	Up	carboxypeptidase E
TCTN3	2.770597	8.94E-05	0.006776	4.198026	Up	tectonic family member 3
YWHAH	2.77017	0.000866	0.02234	3.504563	Up	tyrosine 3-monooxygenase/tryptophan 5-monooxygenase activation protein eta
PLXNA1	2.769986	1.66E-05	0.003137	4.67951	Up	plexin A1
AK1	2.767903	0.000273	0.012251	3.864784	Up	adenylate kinase 1
ORMDL1	2.767548	7.27E-05	0.005968	4.258602	Up	ORMDL sphingolipid biosynthesis regulator 1
SLTM	2.767084	0.000706	0.020072	3.569499	Up	SAFB like transcription modulator
PSMC5	2.760402	0.00097	0.023505	3.468114	Up	proteasome 26S subunit, ATPase 5
UHMK1	2.759638	0.000128	0.008394	4.09178	Up	U2AF homology motif kinase 1
AIFM1	2.757223	7.05E-05	0.005956	4.267206	Up	apoptosis inducing factor mitochondria associated 1
TATDN1	2.756526	0.000672	0.019534	3.585379	Up	TatDDNase domain containing 1
COX6C	2.753983	0.000372	0.014337	3.769771	Up	cytochrome c oxidase subunit 6C
GIPC1	2.753721	0.000335	0.013596	3.802772	Up	GIPC PDZ domain containing family member 1
RPS21	2.753031	0.000158	0.009439	4.030076	Up	ribosomal protein S21
CCDC85B	2.752819	0.001517	0.029361	3.322182	Up	coiled-coil domain containing 85B
UAP1	2.74977	0.001158	0.026017	3.410666	Up	UDP-N-acetylglucosaminepyrophosphorylase 1
POFUT1	2.742386	0.000309	0.012965	3.827384	Up	protein O-fucosyltransferase 1
C3AR1	2.736533	2.1E-05	0.00341	4.613724	Up	complement C3a receptor 1
SRP72	2.734833	5.43E-05	0.005283	4.342922	Up	signal recognition particle 72
ABHD17A	2.734708	0.001052	0.024558	3.441677	Up	abhydrolase domain containing 17A
TOMM7	2.730177	0.001231	0.026634	3.390625	Up	translocase of outer mitochondrial membrane 7
ANP32B	2.728749	0.00161	0.030287	3.302334	Up	acidic nuclear phosphoprotein 32 family member B
TMEM230	2.728085	0.001136	0.025784	3.416807	Up	transmembrane protein 230
NABP2	2.7271	0.000369	0.014306	3.772639	Up	nucleic acid binding protein 2
CDC27	2.726993	0.001334	0.027538	3.364495	Up	cell division cycle 27
FAM20B	2.723814	0.000841	0.021962	3.513811	Up	FAM20B glycosaminoglycan xylosylkinase
ETF1	2.7213	0.000538	0.01724	3.655315	Up	eukaryotic translation termination factor 1
GRHPR	2.720938	0.000857	0.022226	3.507777	Up	glyoxylate and hydroxypyruvatereductase
DERL2	2.720492	0.000154	0.009266	4.038321	Up	derlin 2
RPL36A	2.720487	0.000898	0.02272	3.492922	Up	ribosomal protein L36a
C19orf53	2.718168	0.000499	0.016701	3.678807	Up	chromosome 19 open reading frame 53
GTF2A2	2.716967	0.000389	0.014658	3.756692	Up	general transcription factor IIA subunit 2
GADD45B	2.716143	0.000261	0.011982	3.878655	Up	growth arrest and DNA damage inducible beta
CDIPT	2.714923	0.00013	0.00844	4.088275	Up	CDP-diacylglycerol--inositol 3-phosphatidyltransferase
COX6A1	2.712855	0.000535	0.01724	3.657442	Up	cytochrome c oxidase subunit 6A1
SRSF6	2.711655	0.000694	0.019868	3.575141	Up	serine and arginine rich splicing factor 6
SCARB1	2.709938	0.00054	0.017244	3.654526	Up	scavenger receptor class B member 1
ASAP3	2.709641	3.15E-05	0.004025	4.498522	Up	ArfGAP with SH3 domain, ankyrin repeat and PH domain 3
RNASE1	2.702048	0.00234	0.037096	3.177048	Up	ribonuclease A family member 1, pancreatic
NAPRT	2.701	0.000937	0.023198	3.479213	Up	nicotinatephosphoribosyltransferase
UQCRQ	2.698312	0.000786	0.021056	3.535632	Up	ubiquinol-cytochrome c reductase complex III subunit VII
COX7C	2.697196	0.000888	0.022598	3.496307	Up	cytochrome c oxidase subunit 7C
PSMB1	2.694976	0.000391	0.014705	3.754384	Up	proteasome 20S subunit beta 1
ECH1	2.694774	0.002021	0.034115	3.226513	Up	enoyl-CoA hydratase 1
RPS15A	2.694313	0.000873	0.022405	3.502067	Up	ribosomal protein S15a
ERH	2.693982	9.3E-05	0.006902	4.186545	Up	ERH mRNA splicing and mitosis factor
PSMD3	2.69342	0.000362	0.014114	3.77823	Up	proteasome 26S subunit, non-ATPase 3
MRPS24	2.689672	2.97E-06	0.001776	5.150884	Up	mitochondrial ribosomal protein S24
HSD17B10	2.689486	0.000184	0.010093	3.984586	Up	hydroxysteroid 17-beta dehydrogenase 10
ICMT	2.68742	0.001623	0.030348	3.299776	Up	isoprenylcysteine carboxyl methyltransferase
COX8A	2.68611	0.00082	0.021597	3.521817	Up	cytochrome c oxidase subunit 8A
RPS23	2.683611	0.001311	0.027304	3.370125	Up	ribosomal protein S23
TMEM165	2.683585	0.000309	0.012965	3.827632	Up	transmembrane protein 165
TMEM97	2.683426	1.54E-06	0.001401	5.327084	Up	transmembrane protein 97
COX7A2L	2.680847	0.0015	0.029177	3.325736	Up	cytochrome c oxidase subunit 7A2 like
FAM89B	2.67751	0.003528	0.046326	3.036153	Up	family with sequence similarity 89 member B
SMAD5	2.676775	0.0005	0.016701	3.678519	Up	SMAD family member 5
CCNC	2.675154	0.002737	0.040448	3.123787	Up	cyclin C
CDC42EP2	2.672348	0.000165	0.009593	4.016925	Up	CDC42 effector protein 2
UQCRC1	2.67139	3.96E-05	0.004552	4.433447	Up	ubiquinol-cytochrome c reductase core protein 1
COPS7A	2.671383	0.001825	0.032315	3.260743	Up	COP9 signalosome subunit 7A
CCDC80	2.670347	0.003064	0.042867	3.085018	Up	coiled-coil domain containing 80
PSMB4	2.669349	0.000572	0.017831	3.636053	Up	proteasome 20S subunit beta 4
EIF6	2.664726	0.000227	0.011159	3.920651	Up	eukaryotic translation initiation factor 6
PFDN5	2.657659	0.001117	0.025474	3.422216	Up	prefoldin subunit 5
AFF1	2.657404	0.00036	0.014077	3.78051	Up	AF4/FMR2 family member 1
UQCRFS1	2.656808	0.000434	0.01563	3.722419	Up	ubiquinol-cytochrome c reductase, Rieske iron-sulfur polypeptide 1
TYROBP	2.652926	0.0038	0.047966	3.010176	Up	TYRO protein tyrosine kinase binding protein
ANAPC5	2.650988	0.000463	0.016091	3.702541	Up	anaphase promoting complex subunit 5
TRMT112	2.643049	0.001145	0.025902	3.414206	Up	tRNAmethyltransferase subunit 11-2
ARF5	2.641389	0.002168	0.035481	3.202951	Up	ADP ribosylation factor 5
POLR1D	2.64106	0.000197	0.010419	3.963486	Up	RNA polymerase I and III subunit D
SNX9	2.639558	0.000559	0.017629	3.643451	Up	sorting nexin 9
NDUFS5	2.634939	0.000625	0.018808	3.608112	Up	NADH:ubiquinoneoxidoreductase subunit S5
KDSR	2.634609	0.00089	0.022598	3.495893	Up	3-ketodihydrosphingosine reductase
KRTCAP2	2.634197	0.001854	0.032651	3.255495	Up	keratinocyte associated protein 2
RPAIN	2.632929	0.00075	0.020638	3.55055	Up	RPA interacting protein
OXCT1	2.63204	0.003122	0.043255	3.078538	Up	3-oxoacid CoA-transferase 1
CAPNS1	2.631695	0.000733	0.020454	3.557742	Up	calpain small subunit 1
SERPINH1	2.631035	0.000261	0.011982	3.878996	Up	serpin family H member 1
TNIP2	2.630856	0.000242	0.011614	3.901038	Up	TNFAIP3 interacting protein 2
RASSF3	2.62826	0.004015	0.049293	2.990897	Up	Ras association domain family member 3
RPL27	2.626854	0.001008	0.024036	3.455675	Up	ribosomal protein L27
ARHGAP12	2.624075	0.0007	0.019959	3.572316	Up	Rho GTPase activating protein 12
PRPF31	2.623671	0.000129	0.008399	4.090841	Up	pre-mRNA processing factor 31
KLB	2.623223	0.001365	0.027884	3.356946	Up	klotho beta
TMX4	2.618625	0.00108	0.024945	3.433183	Up	thioredoxin related transmembrane protein 4
NQO2	2.617727	0.002855	0.041527	3.109292	Up	N-ribosyldihydronicotinamide:quinonereductase 2
NME2	2.617495	0.002462	0.038092	3.159908	Up	NME/NM23 nucleoside diphosphate kinase 2
SLC25A26	2.616798	1.27E-05	0.002881	4.754142	Up	solute carrier family 25 member 26
TOMM5	2.615408	0.003222	0.043977	3.067588	Up	translocase of outer mitochondrial membrane 5
ETFB	2.614762	0.001976	0.033674	3.234172	Up	electron transfer flavoprotein subunit beta
MOAP1	2.614239	0.000133	0.008495	4.081606	Up	modulator of apoptosis 1
NACC1	2.613132	1.23E-05	0.002829	4.761618	Up	nucleus accumbens associated 1
NAGLU	2.612492	0.000258	0.011965	3.882494	Up	N-acetyl-alpha-glucosaminidase
XRCC5	2.611431	0.000462	0.016091	3.703217	Up	X-ray repair cross complementing 5
RPL35	2.610712	0.000229	0.011194	3.918588	Up	ribosomal protein L35
KRCC1	2.608947	9.22E-05	0.006864	4.189205	Up	lysine rich coiled-coil 1
PSMB7	2.608925	0.001141	0.025828	3.415417	Up	proteasome 20S subunit beta 7
RPS29	2.608914	0.001176	0.026198	3.405623	Up	ribosomal protein S29
SNRPD2	2.604428	0.000499	0.016701	3.679267	Up	small nuclear ribonucleoprotein D2 polypeptide
RSL24D1	2.604123	0.001206	0.026399	3.397372	Up	ribosomal L24 domain containing 1
RBM3	2.602465	0.000751	0.020661	3.549857	Up	RNA binding motif protein 3
RPL14	2.598328	0.002801	0.041085	3.115761	Up	ribosomal protein L14
DBI	2.593769	0.001225	0.026608	3.392185	Up	diazepam binding inhibitor, acyl-CoA binding protein
RPL13A	2.592976	0.000949	0.023287	3.474966	Up	ribosomal protein L13a
NAB1	2.591638	0.000791	0.021082	3.533288	Up	NGFI-A binding protein 1
STX10	2.590276	0.003718	0.047358	3.01781	Up	syntaxin 10
SLC35E1	2.589777	0.000244	0.011642	3.898916	Up	solute carrier family 35 member E1
GCHFR	2.586356	0.00154	0.029477	3.317136	Up	GTP cyclohydrolase I feedback regulator
WIPI1	2.586044	0.001049	0.024512	3.442577	Up	WD repeat domain, phosphoinositide interacting 1
TMEM50A	2.586036	0.001551	0.029566	3.314827	Up	transmembrane protein 50A
NELFB	2.585911	0.000623	0.018805	3.609156	Up	negative elongation factor complex member B
MRPL9	2.582693	0.000637	0.019003	3.60222	Up	mitochondrial ribosomal protein L9
DIP2C	2.577846	1.51E-06	0.001401	5.333124	Up	disco interacting protein 2 homolog C
PFDN1	2.577843	0.001923	0.033201	3.243123	Up	prefoldin subunit 1
PCYT2	2.574996	0.000343	0.013741	3.795229	Up	phosphate cytidylyltransferase 2, ethanolamine
RPL29	2.574629	0.000911	0.022881	3.488378	Up	ribosomal protein L29
RPL30	2.574242	0.000917	0.022969	3.486043	Up	ribosomal protein L30
EMC4	2.572361	0.00205	0.034257	3.221746	Up	ER membrane protein complex subunit 4
RPL24	2.571676	0.002479	0.038185	3.157525	Up	ribosomal protein L24
RPS15	2.570516	0.001711	0.031119	3.282153	Up	ribosomal protein S15
SMARCD2	2.569052	0.000525	0.017152	3.66321	Up	SWI/SNF related, matrix associated, actin dependent regulator of chromatin, subfamily d, member 2
C9orf78	2.568679	9.48E-05	0.007003	4.180864	Up	chromosome 9 open reading frame 78
BLOC1S1	2.568295	0.001664	0.03072	3.291398	Up	biogenesis of lysosomal organelles complex 1 subunit 1
ATP9A	2.567757	0.000243	0.011614	3.900357	Up	ATPase phospholipid transporting 9A (putative)
MS4A4A	2.566919	5.96E-05	0.005493	4.316129	Up	membrane spanning 4-domains A4A
AFAP1L1	2.565706	0.002187	0.035677	3.199902	Up	actin filament associated protein 1 like 1
CDKN2B	2.565157	0.000599	0.018321	3.621709	Up	cyclin dependent kinase inhibitor 2B
IFI27	2.563118	0.00371	0.047358	3.01863	Up	interferon alpha inducible protein 27
MED4	2.555663	0.001188	0.026311	3.402174	Up	mediator complex subunit 4
DPH5	2.553328	3.71E-05	0.004462	4.452413	Up	diphthamide biosynthesis 5
WIZ	2.550083	0.000617	0.018752	3.612543	Up	WIZ zinc finger
PEX19	2.549485	0.003066	0.042867	3.084727	Up	peroxisomal biogenesis factor 19
RABGEF1	2.547097	0.001793	0.032062	3.266518	Up	RAB guanine nucleotide exchange factor 1
SLC9A3R1	2.544376	0.000906	0.022852	3.490141	Up	SLC9A3 regulator 1
ANO6	2.541247	0.00097	0.023505	3.468073	Up	anoctamin 6
RPLP0	2.539879	0.000147	0.009101	4.051259	Up	ribosomal protein lateral stalk subunit P0
EZH1	2.538446	0.000787	0.021056	3.535293	Up	enhancer of zeste 1 polycomb repressive complex 2 subunit
PSKH1	2.53776	0.001027	0.024288	3.449585	Up	protein serine kinase H1
RPL34	2.53733	0.000646	0.0191	3.597964	Up	ribosomal protein L34
NDUFS4	2.53691	0.000267	0.012096	3.871781	Up	NADH:ubiquinoneoxidoreductase subunit S4
BANF1	2.535865	0.000572	0.017831	3.636063	Up	barrier to autointegration factor 1
MRPL54	2.533785	0.000952	0.023287	3.473973	Up	mitochondrial ribosomal protein L54
CKS1B	2.533771	0.000911	0.022881	3.488195	Up	CDC28 protein kinase regulatory subunit 1B
TMEM184B	2.532583	1.67E-05	0.003137	4.676649	Up	transmembrane protein 184B
PSMD10	2.530765	0.00053	0.01724	3.660242	Up	proteasome 26S subunit, non-ATPase 10
RPS5	2.529965	0.000597	0.018297	3.622831	Up	ribosomal protein S5
CUL4A	2.529656	0.001663	0.03072	3.291653	Up	cullin 4A
MFAP5	2.527961	0.000821	0.021597	3.521542	Up	microfibril associated protein 5
SNTA1	2.526907	0.000372	0.014337	3.770146	Up	syntrophin alpha 1
RPS20	2.52651	0.002839	0.041407	3.111181	Up	ribosomal protein S20
RSL1D1	2.524784	0.000405	0.014978	3.743879	Up	ribosomal L1 domain containing 1
RPS24	2.523665	0.002187	0.035677	3.200019	Up	ribosomal protein S24
ICAM2	2.52328	0.00175	0.031637	3.274701	Up	intercellular adhesion molecule 2
B4GALT2	2.522208	0.001703	0.031013	3.283844	Up	beta-1,4-galactosyltransferase 2
NUDT16	2.521704	0.001545	0.029527	3.315981	Up	nudix hydrolase 16
PFN2	2.519815	0.001062	0.024764	3.438594	Up	profilin 2
RAB10	2.519714	0.003057	0.04281	3.085745	Up	RAB10, member RAS oncogene family
SF3B5	2.519177	0.003604	0.046771	3.028686	Up	splicing factor 3b subunit 5
TCEAL8	2.516685	0.003486	0.04603	3.040293	Up	transcription elongation factor A like 8
CD99L2	2.51621	0.001957	0.03358	3.23728	Up	CD99 molecule like 2
SUCLG1	2.514671	0.001177	0.026198	3.405243	Up	succinate-CoA ligase alpha subunit
LAMA2	2.514019	0.000535	0.01724	3.657403	Up	laminin subunit alpha 2
EIF3E	2.511931	0.000766	0.020887	3.543592	Up	eukaryotic translation initiation factor 3 subunit E
BCL7B	2.511858	0.000361	0.014084	3.779795	Up	BAF chromatin remodeling complex subunit BCL7B
SEPHS2	2.511732	0.000202	0.010561	3.955986	Up	selenophosphatesynthetase 2
LOXL2	2.511606	0.000883	0.022598	3.498175	Up	lysyl oxidase like 2
PMM1	2.511461	0.00143	0.02855	3.341501	Up	phosphomannomutase 1
DNPH1	2.50873	0.002905	0.041811	3.103319	Up	2'-deoxynucleoside 5'-phosphate N-hydrolase 1
RARRES2	2.504265	0.003791	0.047902	3.011021	Up	retinoic acid receptor responder 2
COA3	2.50271	0.001102	0.025262	3.426827	Up	cytochrome c oxidase assembly factor 3
MRPS34	2.502001	0.000919	0.022981	3.485574	Up	mitochondrial ribosomal protein S34
SLC30A7	-3.56691	4.14E-07	0.00095	-5.67461	Down	solute carrier family 30 member 7
VTI1A	-3.31043	1.05E-07	0.000478	-6.03155	Down	vesicle transport through interaction with t-SNAREs 1A
ERG	-3.30133	2.32E-05	0.003497	-4.58491	Down	ETS transcription factor ERG
ZFYVE1	-3.18784	1.03E-06	0.001179	-5.43378	Down	zinc finger FYVE-type containing 1
AAK1	-3.1737	2.73E-06	0.001776	-5.17359	Down	AP2 associated kinase 1
URB2	-3.16731	8.19E-07	0.00108	-5.49481	Down	URB2 ribosome biogenesis homolog
IGIP	-3.12032	5.55E-05	0.005363	-4.33643	Down	IgA inducing protein
ITIH4	-2.9168	3.51E-06	0.001852	-5.10571	Down	inter-alpha-trypsin inhibitor heavy chain 4
LOC646214	-2.85088	0.000346	0.013805	-3.79234	Down	p21 protein (Cdc42/Rac)-activated kinase 2 pseudogene
ZNF486	-2.84793	1.14E-05	0.002738	-4.78297	Down	zinc finger protein 486
TET3	-2.81596	1.11E-06	0.001179	-5.41343	Down	tetmethylcytosinedioxygenase 3
HECW2	-2.8127	3.17E-05	0.004025	-4.49639	Down	HECT, C2 and WW domain containing E3 ubiquitin protein ligase 2
LPXN	-2.80145	3.07E-06	0.001776	-5.14246	Down	leupaxin
GOSR1	-2.79883	1.73E-05	0.003174	-4.66687	Down	golgi SNAP receptor complex member 1
PDPR	-2.78381	4.99E-05	0.005058	-4.36736	Down	pyruvate dehydrogenase phosphatase regulatory subunit
TNKS	-2.74917	9.17E-05	0.006848	-4.19071	Down	tankyrase
ECD	-2.73445	0.000348	0.013823	-3.79107	Down	ecdysoneless cell cycle regulator
ABCC5	-2.72387	1.16E-05	0.002752	-4.77862	Down	ATP binding cassette subfamily C member 5
STXBP5	-2.71632	0.000176	0.009858	-3.99744	Down	syntaxin binding protein 5
WDR19	-2.69898	0.000615	0.018721	-3.61344	Down	WD repeat domain 19
SARM1	-2.69717	1.19E-09	1.98E-05	-7.17529	Down	sterile alpha and TIR motif containing 1
TRAF5	-2.66677	0.000107	0.007492	-4.14579	Down	TNF receptor associated factor 5
SYNE2	-2.663	5.87E-05	0.005493	-4.32047	Down	spectrin repeat containing nuclear envelope protein 2
CFP	-2.6567	0.000675	0.019585	-3.58386	Down	complement factor properdin
ANKRD20A9P	-2.64322	0.001714	0.031148	-3.28161	Down	ankyrin repeat domain 20 family member A9, pseudogene
FAT4	-2.63099	0.000494	0.016631	-3.68187	Down	FAT atypical cadherin 4
HIVEP1	-2.62581	8.18E-05	0.006387	-4.22422	Down	HIVEP zinc finger 1
SNAP47	-2.61436	0.000216	0.010911	-3.93599	Down	synaptosome associated protein 47
DMRT2	-2.61357	0.000161	0.009544	-4.02349	Down	doublesex and mab-3 related transcription factor 2
GEMIN5	-2.60553	3.51E-05	0.004269	-4.46757	Down	gem nuclear organelle associated protein 5
LOC643406	-2.59315	0.000435	0.015643	-3.72139	Down	uncharacterized LOC643406
OBSCN	-2.58615	0.000237	0.011453	-3.90744	Down	obscurin, cytoskeletal calmodulin and titin-interacting RhoGEF
ATP13A1	-2.57902	6.26E-05	0.005556	-4.3019	Down	ATPase 13A1
LOC105372795	-2.56184	2.46E-06	0.001776	-5.2015	Down	uncharacterized LOC105372795
PXMP4	-2.55289	0.000508	0.016883	-3.67316	Down	peroxisomal membrane protein 4
FAS	-2.51889	0.000221	0.011049	-3.92874	Down	Fas cell surface death receptor
MGAM	-2.5179	0.001226	0.026608	-3.39213	Down	maltase-glucoamylase
ERCC6L2	-2.49433	0.000462	0.016091	-3.70269	Down	ERCC excision repair 6 like 2
MFAP3	-2.48427	9.78E-05	0.00709	-4.17183	Down	microfibril associated protein 3
TPD52	-2.48206	0.001319	0.027418	-3.36809	Down	tumor protein D52
PPP1R3B	-2.47428	0.001536	0.029477	-3.31797	Down	protein phosphatase 1 regulatory subunit 3B
PTGDR	-2.46695	4.33E-07	0.00095	-5.66312	Down	prostaglandin D2 receptor
AHSP	-2.45099	4.99E-06	0.002061	-5.01026	Down	alpha hemoglobin stabilizing protein
ODF2	-2.44884	0.000531	0.01724	-3.65942	Down	outer dense fiber of sperm tails 2
MAU2	-2.44852	0.002675	0.039958	-3.1315	Down	MAU2 sister chromatid cohesion factor
PTOV1-AS2	-2.44504	8.73E-06	0.002428	-4.85721	Down	PTOV1 antisense RNA 2
SLCO4A1	-2.42489	9.04E-05	0.006791	-4.19486	Down	solute carrier organic anion transporter family member 4A1
RAP1GAP2	-2.41727	0.000428	0.015491	-3.72656	Down	RAP1 GTPase activating protein 2
GUSBP11	-2.41706	1.72E-05	0.003165	-4.66966	Down	GUSB pseudogene 11
CHST14	-2.40289	6.43E-06	0.002175	-4.94093	Down	carbohydrate sulfotransferase 14
SMYD4	-2.3923	0.000889	0.022598	-3.49612	Down	SET and MYND domain containing 4
CACNA2D4	-2.38689	1.62E-05	0.003137	-4.6856	Down	calcium voltage-gated channel auxiliary subunit alpha2delta 4
ERO1A	-2.3862	0.001273	0.026966	-3.37975	Down	endoplasmic reticulum oxidoreductase 1 alpha
ATRNL1	-2.37103	1.84E-05	0.003308	-4.64955	Down	attractin like 1
ATL1	-2.37076	0.000548	0.017445	-3.64947	Down	atlastinGTPase 1
EPHB4	-2.36058	0.000451	0.015882	-3.71047	Down	EPH receptor B4
AOC4P	-2.35367	3.9E-06	0.001884	-5.07705	Down	amine oxidase copper containing 4, pseudogene
SLC25A13	-2.34818	0.000134	0.008515	-4.07881	Down	solute carrier family 25 member 13
FAM13B	-2.34187	0.003334	0.044854	-3.05577	Down	family with sequence similarity 13 member B
TRAK1	-2.33457	0.000883	0.022598	-3.49834	Down	trafficking kinesin protein 1
ABI3	-2.3343	0.000274	0.012278	-3.8636	Down	ABI family member 3
SEMA6B	-2.33392	4.57E-05	0.004952	-4.39248	Down	semaphorin 6B
METTL21A	-2.33362	0.002789	0.040954	-3.11724	Down	methyltransferase like 21A
ZNF778	-2.33087	3.93E-06	0.001884	-5.07521	Down	zinc finger protein 778
PDE4B	-2.32078	0.000786	0.021056	-3.5357	Down	phosphodiesterase 4B
NDC1	-2.31958	0.000208	0.010631	-3.94732	Down	NDC1 transmembranenucleoporin
SLC9A7	-2.31311	0.001151	0.025956	-3.41266	Down	solute carrier family 9 member A7
NLGN2	-2.31264	7.89E-05	0.006274	-4.23441	Down	neuroligin 2
IKZF4	-2.30922	0.000113	0.007788	-4.12818	Down	IKAROS family zinc finger 4
MAST3	-2.3067	0.001042	0.024428	-3.44474	Down	microtubule associated serine/threonine kinase 3
SAP25	-2.30632	0.000324	0.013394	-3.81298	Down	Sin3A associated protein 25
ZNF213-AS1	-2.30592	3.03E-06	0.001776	-5.14592	Down	ZNF213 antisense RNA 1 (head to head)
ADAMTS13	-2.30408	2.03E-06	0.001623	-5.25359	Down	ADAM metallopeptidase with thrombospondin type 1 motif 13
CDH19	-2.30007	9.85E-06	0.002623	-4.82394	Down	cadherin 19
DOCK9	-2.28015	0.002517	0.038469	-3.15239	Down	dedicator of cytokinesis 9
PARP10	-2.27632	0.003654	0.047012	-3.0239	Down	poly (ADP-ribose) polymerase family member 10
LOC648987	-2.27592	2.87E-06	0.001776	-5.16	Down	uncharacterized LOC648987
GIMAP8	-2.27461	0.001512	0.029333	-3.32309	Down	GTPase, IMAP family member 8
COL8A1	-2.27216	0.00307	0.042867	-3.08427	Down	collagen type VIII alpha 1 chain
MYO7A	-2.26997	5.89E-06	0.002131	-4.96524	Down	myosin VIIA
SLC25A35	-2.26394	4.18E-05	0.004645	-4.41821	Down	solute carrier family 25 member 35
ESR1	-2.25478	0.000772	0.020954	-3.54124	Down	estrogen receptor 1
FAM71F2	-2.25074	5.55E-06	0.002118	-4.98153	Down	family with sequence similarity 71 member F2
ZNF493	-2.24829	0.000671	0.019525	-3.58587	Down	zinc finger protein 493
CEP135	-2.24319	1.89E-05	0.003342	-4.64292	Down	centrosomal protein 135
CEP126	-2.24051	0.001032	0.024341	-3.44789	Down	centrosomal protein 126
ASTN2	-2.23592	0.001156	0.026012	-3.41115	Down	astrotactin 2
IPP	-2.22612	0.000101	0.007163	-4.16214	Down	intracisternal A particle-promoted polypeptide
TANGO6	-2.2248	0.000418	0.015262	-3.73417	Down	transport and golgi organization 6 homolog
ZXDC	-2.22107	0.001926	0.033211	-3.24265	Down	ZXD family zinc finger C
TMCO4	-2.21386	0.000912	0.022891	-3.48775	Down	transmembrane and coiled-coil domains 4
WSCD1	-2.20938	1.36E-05	0.003004	-4.73545	Down	WSC domain containing 1
PLXNB3	-2.20331	0.000194	0.010359	-3.96765	Down	plexin B3
SPAST	-2.19723	0.003713	0.047358	-3.01835	Down	spastin
LRSAM1	-2.19647	0.00028	0.012403	-3.85725	Down	leucine rich repeat and sterile alpha motif containing 1
BANP	-2.19578	0.002678	0.039958	-3.13116	Down	BTG3 associated nuclear protein
SCAF4	-2.1951	0.001471	0.028954	-3.33217	Down	SR-related CTD associated factor 4
SDCCAG8	-2.19392	0.001272	0.026966	-3.38007	Down	serologically defined colon cancer antigen 8
KCNQ1OT1	-2.19178	0.002417	0.037819	-3.16609	Down	KCNQ1 opposite strand/antisense transcript 1
CD82	-2.18167	0.001254	0.026814	-3.38461	Down	CD82 molecule
KIAA0754	-2.17697	0.000994	0.023761	-3.46018	Down	KIAA0754
PTPRN2	-2.17646	0.00099	0.023704	-3.46132	Down	protein tyrosine phosphatase receptor type N2
MYLK-AS1	-2.16901	0.000153	0.009259	-4.03959	Down	MYLK antisense RNA 1
PLXDC1	-2.16377	0.001307	0.027262	-3.37099	Down	plexin domain containing 1
TDRD5	-2.15738	4.87E-05	0.005015	-4.37413	Down	tudor domain containing 5
LCAT	-2.15173	0.000278	0.012383	-3.85932	Down	lecithin-cholesterol acyltransferase
PRDM11	-2.15109	6.78E-07	0.001025	-5.54474	Down	PR/SET domain 11
RASIP1	-2.14799	0.000745	0.02059	-3.55262	Down	Ras interacting protein 1
SPN	-2.14456	0.002682	0.039958	-3.13065	Down	sialophorin
SHISA9	-2.14372	0.00386	0.048313	-3.0047	Down	shisa family member 9
ANKRD6	-2.14267	0.003628	0.046875	-3.0264	Down	ankyrin repeat domain 6
MAK	-2.13779	2.41E-05	0.003515	-4.57405	Down	male germ cell associated kinase
EPHA4	-2.13565	0.000848	0.022087	-3.51135	Down	EPH receptor A4
UBR5-AS1	-2.12673	2.81E-05	0.003842	-4.53078	Down	UBR5 antisense RNA 1
CTC1	-2.12661	0.003144	0.043426	-3.07609	Down	CST telomere replication complex component 1
NPHP4	-2.12045	7.65E-05	0.006152	-4.24347	Down	nephrocystin 4
RFX3	-2.11961	0.000286	0.012464	-3.85121	Down	regulatory factor X3
PLD1	-2.10857	0.000478	0.016343	-3.69227	Down	phospholipase D1
ZNF474	-2.10767	3.19E-06	0.001776	-5.13193	Down	zinc finger protein 474
LCK	-2.10338	0.000224	0.011092	-3.92537	Down	LCK proto-oncogene, Src family tyrosine kinase
ZCCHC4	-2.10229	0.002042	0.034193	-3.22302	Down	zinc finger CCHC-type containing 4
PRPS2	-2.09872	0.001319	0.027418	-3.3681	Down	phosphoribosyl pyrophosphate synthetase 2
TAF4B	-2.09126	0.000171	0.009664	-4.00627	Down	TATA-box binding protein associated factor 4b
CDC42BPA	-2.08947	0.001389	0.028155	-3.3511	Down	CDC42 binding protein kinase alpha
LINC01230	-2.0891	0.00033	0.013567	-3.80731	Down	long intergenic non-protein coding RNA 1230
CPAMD8	-2.08813	0.000302	0.012829	-3.83385	Down	C3 and PZP like alpha-2-macroglobulin domain containing 8
PARP9	-2.08698	0.001921	0.033188	-3.24363	Down	poly (ADP-ribose) polymerase family member 9
ZFYVE27	-2.08654	0.000479	0.016343	-3.69181	Down	zinc finger FYVE-type containing 27
IWS1	-2.08516	0.002881	0.041667	-3.10619	Down	interacts with SUPT6H, CTD assembly factor 1
MMRN1	-2.08143	0.000451	0.015882	-3.7104	Down	multimerin 1
ST20-AS1	-2.07521	6.76E-06	0.00223	-4.92723	Down	ST20 antisense RNA 1
CALCR	-2.07464	5.03E-05	0.005083	-4.36486	Down	calcitonin receptor
KAT7	-2.06821	0.003226	0.043983	-3.06718	Down	lysine acetyltransferase 7
ARHGAP22	-2.05873	4.78E-06	0.002061	-5.02181	Down	Rho GTPase activating protein 22
TMEM199	-2.05777	0.002257	0.036467	-3.18933	Down	transmembrane protein 199
NTN1	-2.05766	0.000168	0.009643	-4.01109	Down	netrin 1
LAMA5	-2.05607	0.002141	0.035221	-3.20706	Down	laminin subunit alpha 5
SPNS3	-2.05195	2.18E-05	0.00341	-4.60239	Down	sphingolipid transporter 3 (putative)
NEDD4L	-2.04969	0.001542	0.029477	-3.31678	Down	NEDD4 like E3 ubiquitin protein ligase
BSN	-2.04855	2.9E-05	0.003871	-4.52186	Down	bassoon presynaptic cytomatrix protein
TMED8	-2.04722	7.63E-06	0.002243	-4.89431	Down	transmembrane p24 trafficking protein family member 8
STRBP	-2.04479	0.003521	0.046281	-3.03684	Down	spermatid perinuclear RNA binding protein
ATP13A4	-2.04282	5.91E-05	0.005493	-4.3185	Down	ATPase 13A4
KLC2	-2.03984	0.001017	0.024181	-3.45276	Down	kinesin light chain 2
RLF	-2.03665	0.001889	0.032833	-3.2492	Down	rearranged L-myc fusion
TTF1	-2.03264	0.002608	0.03937	-3.14019	Down	transcription termination factor 1
TMEM79	-2.0311	5.41E-05	0.005279	-4.34419	Down	transmembrane protein 79
ZCCHC2	-2.02905	0.002297	0.036735	-3.18334	Down	zinc finger CCHC-type containing 2
ANKRD13B	-2.02722	0.001008	0.024036	-3.45558	Down	ankyrin repeat domain 13B
IFT122	-2.02015	0.002868	0.041571	-3.10772	Down	intraflagellar transport 122
MAP 3K14	-2.01534	0.003484	0.046026	-3.0405	Down	mitogen-activated protein kinase kinasekinase 14
SPATA6L	-2.01191	3.29E-05	0.004116	-4.48594	Down	spermatogenesis associated 6 like
SCOC-AS1	-2.01128	0.00093	0.023147	-3.48171	Down	SCOC antisense RNA 1
LMO7	-2.00579	0.000573	0.017831	-3.63573	Down	LIM domain 7
MEX3B	-1.99674	2.01E-05	0.00341	-4.6259	Down	mex-3 RNA binding family member B
IQCK	-1.99382	0.00025	0.011782	-3.89149	Down	IQ motif containing K
GPATCH1	-1.9934	0.000126	0.008296	-4.09746	Down	G-patch domain containing 1
HPS4	-1.98942	0.002434	0.037832	-3.16378	Down	HPS4 biogenesis of lysosomal organelles complex 3 subunit 2
FGFR3	-1.98496	1.56E-05	0.003137	-4.6955	Down	fibroblast growth factor receptor 3
GABRA4	-1.98306	0.000116	0.007921	-4.1216	Down	gamma-aminobutyric acid type A receptor alpha4 subunit
TMEM116	-1.97917	0.000648	0.0191	-3.5967	Down	transmembrane protein 116
CTU1	-1.97326	4.92E-06	0.002061	-5.01408	Down	cytosolic thiouridylase subunit 1
PPIEL	-1.9687	0.002144	0.035221	-3.20669	Down	peptidylprolylisomerase E like pseudogene
FUT11	-1.965	0.000688	0.019806	-3.57787	Down	fucosyltransferase 11
TOP3A	-1.96066	0.00295	0.04205	-3.098	Down	DNA topoisomerase III alpha
C22orf34	-1.95954	0.000251	0.011782	-3.89083	Down	chromosome 22 open reading frame 34
ASIC3	-1.95737	4.65E-05	0.005	-4.38764	Down	acid sensing ion channel subunit 3
SNPH	-1.95235	0.002152	0.035284	-3.20546	Down	syntaphilin
ZNF547	-1.95217	0.000487	0.016474	-3.68677	Down	zinc finger protein 547
FEZ1	-1.94468	0.000873	0.022405	-3.50206	Down	fasciculation and elongation protein zeta 1
ENTPD5	-1.94339	0.000264	0.012058	-3.87463	Down	ectonucleoside triphosphate diphosphohydrolase 5 (inactive)
LOC729683	-1.94235	2.6E-05	0.00365	-4.55269	Down	uncharacterized LOC729683
S1PR5	-1.94099	8.19E-05	0.006387	-4.22381	Down	sphingosine-1-phosphate receptor 5
ZNF300P1	-1.93492	0.000171	0.009664	-4.00596	Down	zinc finger protein 300 pseudogene 1
UNC13C	-1.93485	0.000438	0.015649	-3.7196	Down	unc-13 homolog C
LOC101928370	-1.93447	2.11E-06	0.001634	-5.24334	Down	uncharacterized LOC101928370
ZNF555	-1.92846	0.000376	0.014411	-3.76684	Down	zinc finger protein 555
GADD45G	-1.92497	0.001994	0.03385	-3.23101	Down	growth arrest and DNA damage inducible gamma
KCNC1	-1.92247	2.19E-05	0.00341	-4.60096	Down	potassium voltage-gated channel subfamily C member 1
CARD14	-1.91822	6.28E-06	0.002168	-4.9474	Down	caspase recruitment domain family member 14
STK36	-1.91359	0.003625	0.046875	-3.0267	Down	serine/threonine kinase 36
TRIB3	-1.91164	0.00031	0.012992	-3.82577	Down	tribblespseudokinase 3
ZNF124	-1.9092	0.000271	0.012156	-3.86767	Down	zinc finger protein 124
MPZL3	-1.90884	0.000298	0.012703	-3.83854	Down	myelin protein zero like 3
TOR4A	-1.90324	0.00029	0.012546	-3.84663	Down	torsin family 4 member A
MIR3916	-1.90072	4.48E-06	0.002039	-5.03938	Down	microRNA 3916
ZNF334	-1.89919	6.18E-06	0.002168	-4.9521	Down	zinc finger protein 334
TTLL3	-1.89535	0.000105	0.007409	-4.1499	Down	tubulin tyrosine ligase like 3
ZKSCAN3	-1.89411	0.001595	0.030071	-3.30542	Down	zinc finger with KRAB and SCAN domains 3
PAPLN	-1.88842	0.001527	0.029477	-3.31986	Down	papilin, proteoglycan like sulfated glycoprotein
KNTC1	-1.88703	0.000207	0.01061	-3.94852	Down	kinetochore associated 1
SLC1A7	-1.88316	0.000163	0.009559	-4.01961	Down	solute carrier family 1 member 7
BCL9	-1.87682	0.00158	0.029935	-3.30857	Down	BCL9 transcription coactivator
ZNF496	-1.87502	0.00041	0.015068	-3.74029	Down	zinc finger protein 496
LNX2	-1.87272	0.003479	0.045998	-3.04098	Down	ligand of numb-protein X 2
DNAJC18	-1.8714	0.000203	0.010561	-3.95515	Down	DnaJ heat shock protein family (Hsp40) member C18
ZNF114	-1.86888	6.34E-05	0.0056	-4.29807	Down	zinc finger protein 114
VWA3B	-1.86543	3.01E-05	0.003964	-4.51187	Down	von Willebrand factor A domain containing 3B
ARNTL2	-1.86522	0.002029	0.034127	-3.22522	Down	aryl hydrocarbon receptor nuclear translocator like 2
NGFR	-1.85434	0.000295	0.012696	-3.84106	Down	nerve growth factor receptor
CLEC1A	-1.85306	0.001166	0.026095	-3.40829	Down	C-type lectin domain family 1 member A
ZNF687	-1.85305	0.003658	0.047012	-3.02347	Down	zinc finger protein 687
ZNF69	-1.85271	2.47E-05	0.003545	-4.56704	Down	zinc finger protein 69
MTF1	-1.85038	0.000974	0.023505	-3.46684	Down	metal regulatory transcription factor 1
ZNF154	-1.84509	0.00012	0.008053	-4.11197	Down	zinc finger protein 154
SLAMF6	-1.84139	0.00019	0.010241	-3.9745	Down	SLAM family member 6
TMEM255B	-1.8379	5.28E-05	0.005263	-4.35097	Down	transmembrane protein 255B
HLA-H	-1.83482	0.002773	0.040755	-3.11929	Down	major histocompatibility complex, class I, H (pseudogene)
KBTBD7	-1.82918	0.000254	0.011836	-3.88686	Down	kelch repeat and BTB domain containing 7
CDC20B	-1.82896	6.91E-05	0.005946	-4.2731	Down	cell division cycle 20B
SLC44A5	-1.82893	1.48E-05	0.003137	-4.71193	Down	solute carrier family 44 member 5
MEN1	-1.8227	0.001593	0.030071	-3.30582	Down	menin 1
SCNN1D	-1.82156	0.003912	0.048656	-3.00003	Down	sodium channel epithelial 1 delta subunit
PFAS	-1.81549	0.002896	0.041776	-3.10436	Down	phosphoribosylformylglycinamidine synthase
EVA1C	-1.81455	0.001419	0.028438	-3.34407	Down	eva-1 homolog C
TIAM2	-1.80764	6.94E-05	0.005946	-4.27171	Down	TIAM Rac1 associated GEF 2
HLA-F-AS1	-1.80482	0.002916	0.041853	-3.10204	Down	HLA-F antisense RNA 1
TRAPPC9	-1.80093	0.00091	0.022881	-3.48851	Down	trafficking protein particle complex 9
APCDD1L	-1.80071	1.64E-05	0.003137	-4.68254	Down	APC down-regulated 1 like
CYTIP	-1.79864	0.001215	0.026482	-3.39492	Down	cytohesin 1 interacting protein
NUTM1	-1.79716	2.14E-05	0.00341	-4.60821	Down	NUT midline carcinoma family member 1
ARHGAP39	-1.79263	0.000319	0.013245	-3.81744	Down	Rho GTPase activating protein 39
SPTB	-1.79073	0.001482	0.028982	-3.32988	Down	spectrin beta, erythrocytic
ZNF469	-1.78919	7.59E-05	0.006143	-4.24571	Down	zinc finger protein 469
AZIN1-AS1	-1.78541	1.92E-05	0.003347	-4.63776	Down	AZIN1 antisense RNA 1
BCL2L11	-1.78395	0.001862	0.032689	-3.25392	Down	BCL2 like 11
CLDN1	-1.78076	0.000331	0.013573	-3.80619	Down	claudin 1
ZNF836	-1.77956	6.7E-06	0.00223	-4.92984	Down	zinc finger protein 836
APBA1	-1.77749	0.002859	0.041537	-3.10878	Down	amyloid beta precursor protein binding family A member 1
C8orf37	-1.77616	0.000155	0.009321	-4.03521	Down	chromosome 8 open reading frame 37
LOC391322	-1.77531	9.16E-06	0.00249	-4.84394	Down	D-dopachrometautomerase-like
SPATA2L	-1.77449	0.002137	0.035219	-3.2077	Down	spermatogenesis associated 2 like
GLIS1	-1.7719	2.07E-05	0.00341	-4.61666	Down	GLIS family zinc finger 1
LOC389765	-1.76734	7.38E-07	0.001025	-5.5224	Down	kinesin family member 27 pseudogene
DENND2C	-1.76558	1.69E-05	0.003137	-4.67437	Down	DENN domain containing 2C
MFAP3L	-1.76481	0.003083	0.042962	-3.08279	Down	microfibril associated protein 3 like
TREML1	-1.76402	5.96E-05	0.005493	-4.31615	Down	triggering receptor expressed on myeloid cells like 1
DNHD1	-1.76398	0.001285	0.027046	-3.37662	Down	dynein heavy chain domain 1
SETD6	-1.75933	5.78E-05	0.005462	-4.32489	Down	SET domain containing 6, protein lysine methyltransferase
TTC34	-1.75712	4.74E-05	0.005	-4.38193	Down	tetratricopeptide repeat domain 34
SARDH	-1.74727	0.000444	0.015767	-3.71564	Down	sarcosine dehydrogenase
ZNF385C	-1.74659	5.06E-06	0.002061	-5.00645	Down	zinc finger protein 385C
NEXN-AS1	-1.74174	0.001791	0.03205	-3.26688	Down	NEXN antisense RNA 1
CDHR3	-1.741	5.08E-05	0.00511	-4.36224	Down	cadherin related family member 3
SPRYD4	-1.7362	0.00381	0.048018	-3.00934	Down	SPRY domain containing 4
ZSCAN25	-1.73514	0.000224	0.011092	-3.92529	Down	zinc finger and SCAN domain containing 25
FAM157C	-1.73405	3.95E-05	0.004552	-4.43393	Down	family with sequence similarity 157 member C
ARSG	-1.73192	0.000264	0.012045	-3.8757	Down	arylsulfatase G
GLI2	-1.72398	8.75E-06	0.002428	-4.85662	Down	GLI family zinc finger 2
NSUN7	-1.72112	0.000383	0.014539	-3.76087	Down	NOP2/Sun RNA methyltransferase family member 7
BMP3	-1.71974	0.00051	0.016932	-3.67185	Down	bone morphogenetic protein 3
PPT2	-1.71813	1.01E-05	0.002625	-4.81647	Down	palmitoyl-protein thioesterase 2
CCNJL	-1.7168	0.00297	0.042241	-3.0957	Down	cyclin J like
C3orf70	-1.71506	1.17E-05	0.002752	-4.77652	Down	chromosome 3 open reading frame 70
FBF1	-1.71426	0.002996	0.042479	-3.09265	Down	Fas binding factor 1
SEC14L2	-1.70866	7.5E-05	0.006105	-4.24929	Down	SEC14 like lipid binding 2
YPEL4	-1.70708	0.000495	0.016631	-3.68141	Down	yippee like 4
PCDH11Y	-1.70054	0.00111	0.025344	-3.42438	Down	protocadherin 11 Y-linked
AFAP1L2	-1.68648	4.19E-05	0.004645	-4.41718	Down	actin filament associated protein 1 like 2
ZNF674-AS1	-1.6796	0.001198	0.026347	-3.39966	Down	ZNF674 antisense RNA 1 (head to head)
KCNQ4	-1.67351	1.12E-05	0.002709	-4.78912	Down	potassium voltage-gated channel subfamily Q member 4
SULT1B1	-1.67126	9.73E-05	0.00709	-4.17312	Down	sulfotransferase family 1B member 1
MORN1	-1.66843	0.000538	0.01724	-3.6552	Down	MORN repeat containing 1
PCDH17	-1.66757	7.41E-05	0.006068	-4.25287	Down	protocadherin 17
FOXP2	-1.66727	0.000941	0.023272	-3.47788	Down	forkhead box P2
XYLB	-1.66722	0.001703	0.031013	-3.28371	Down	xylulokinase
CTNNA3	-1.66694	0.000163	0.009544	-4.02144	Down	catenin alpha 3
NLRP6	-1.66633	0.000524	0.017152	-3.66377	Down	NLR family pyrin domain containing 6
SLC16A13	-1.66401	0.000624	0.018805	-3.60875	Down	solute carrier family 16 member 13
GLI3	-1.6638	0.003245	0.044179	-3.06511	Down	GLI family zinc finger 3
SYNJ2	-1.66358	0.000209	0.010651	-3.94618	Down	synaptojanin 2
C2orf15	-1.6617	0.000283	0.012404	-3.85419	Down	chromosome 2 open reading frame 15
SCIMP	-1.65716	0.002226	0.036099	-3.19403	Down	SLP adaptor and CSK interacting membrane protein
KIR2DL4	-1.6566	0.000227	0.01115	-3.92146	Down	killer cell immunoglobulin like receptor, two Ig domains and long cytoplasmic tail 4
FHL3	-1.65441	0.00305	0.042759	-3.08659	Down	four and a half LIM domains 3
OVGP1	-1.65215	1.12E-06	0.001179	-5.41243	Down	oviductal glycoprotein 1
N4BP3	-1.65201	0.000581	0.017987	-3.63113	Down	NEDD4 binding protein 3
ELOVL7	-1.64755	3.88E-05	0.00453	-4.43907	Down	ELOVL fatty acid elongase 7
ESRG	-1.64744	0.001428	0.028535	-3.34192	Down	embryonic stem cell related
ACTA2-AS1	-1.64552	0.000128	0.008394	-4.09176	Down	ACTA2 antisense RNA 1
CNKSR3	-1.64218	7.19E-05	0.005962	-4.26146	Down	CNKSR family member 3
ZNF347	-1.63729	0.002754	0.040644	-3.12164	Down	zinc finger protein 347
SGMS1-AS1	-1.6362	3.3E-06	0.001776	-5.12249	Down	SGMS1 antisense RNA 1
ZKSCAN2	-1.63535	4.67E-05	0.005	-4.38616	Down	zinc finger with KRAB and SCAN domains 2
AMIGO1	-1.63486	0.000936	0.023198	-3.47947	Down	adhesion molecule with Ig like domain 1
ZC3H10	-1.63262	0.001463	0.028876	-3.33409	Down	zinc finger CCCH-type containing 10
MUC17	-1.62593	0.000109	0.007553	-4.14108	Down	mucin 17, cell surface associated
ZNF559-ZNF177	-1.62399	6.21E-05	0.005556	-4.30427	Down	ZNF559-ZNF177 readthrough
NUTM2D	-1.62355	0.000228	0.011159	-3.92008	Down	NUT family member 2D
ABCB4	-1.62247	3.99E-06	0.001884	-5.07099	Down	ATP binding cassette subfamily B member 4
LOC100652768	-1.61749	0.000307	0.012935	-3.82956	Down	uncharacterized LOC100652768
C8orf58	-1.61509	5.62E-06	0.002118	-4.97786	Down	chromosome 8 open reading frame 58
WRAP73	-1.61325	4.07E-06	0.001884	-5.06554	Down	WD repeat containing, antisense to TP73
SLC24A4	-1.61174	2.06E-05	0.00341	-4.61911	Down	solute carrier family 24 member 4
LENG8-AS1	-1.61071	2.39E-05	0.003497	-4.57709	Down	LENG8 antisense RNA 1
C2orf66	-1.60563	1.03E-05	0.002643	-4.81096	Down	chromosome 2 open reading frame 66
SMIM10L2A	-1.60523	9.09E-05	0.006806	-4.19335	Down	small integral membrane protein 10 like 2A
CYP1B1-AS1	-1.60433	0.000107	0.007513	-4.14419	Down	CYP1B1 antisense RNA 1
CTNND1	-1.60088	0.000109	0.007568	-4.13895	Down	catenin delta 1
ADCY10P1	-1.60028	4.19E-05	0.004645	-4.4176	Down	ADCY10 pseudogene 1
LOC100130298	-1.59936	3.39E-05	0.004196	-4.47779	Down	hCG1816373-like
FLJ42627	-1.59816	0.001285	0.027046	-3.37677	Down	uncharacterized LOC645644
CRISPLD1	-1.59815	0.000698	0.019931	-3.57312	Down	cysteine rich secretory protein LCCL domain containing 1
MESTIT1	-1.59755	1.51E-06	0.001401	-5.33225	Down	MEST intronic transcript 1, antisense RNA
ASPG	-1.59743	0.001369	0.027894	-3.35594	Down	asparaginase
PRICKLE2-AS1	-1.59631	0.000336	0.013596	-3.80182	Down	PRICKLE2 antisense RNA 1
LINC00865	-1.59557	2.66E-05	0.00371	-4.54615	Down	long intergenic non-protein coding RNA 865
LINC01001	-1.59556	1.77E-06	0.001545	-5.29009	Down	long intergenic non-protein coding RNA 1001
GRK4	-1.59417	1.69E-05	0.003137	-4.67411	Down	G protein-coupled receptor kinase 4
ZNF300	-1.59305	5.6E-05	0.005391	-4.33388	Down	zinc finger protein 300
CADM3-AS1	-1.59265	7.19E-05	0.005962	-4.26163	Down	CADM3 antisense RNA 1
SIT1	-1.59069	0.000397	0.014772	-3.75003	Down	signaling threshold regulating transmembrane adaptor 1
LINC01138	-1.59045	0.001479	0.028971	-3.33042	Down	long intergenic non-protein coding RNA 1138
DNAH5	-1.5878	0.000268	0.012109	-3.87093	Down	dynein axonemal heavy chain 5
FAM155B	-1.58584	2.87E-05	0.003871	-4.52525	Down	family with sequence similarity 155 member B
SLC5A4	-1.58234	3.27E-06	0.001776	-5.12479	Down	solute carrier family 5 member 4
ZNF573	-1.58059	6.33E-06	0.002168	-4.94541	Down	zinc finger protein 573
RCAN3	-1.5793	2.89E-05	0.003871	-4.52291	Down	RCAN family member 3
DNAH3	-1.57822	0.000296	0.012696	-3.83989	Down	dynein axonemal heavy chain 3
UBXN10	-1.57771	1.9E-05	0.003343	-4.64092	Down	UBX domain protein 10
CLEC4A	-1.57555	0.003924	0.048741	-2.99895	Down	C-type lectin domain family 4 member A
RFFL	-1.57423	0.000171	0.009664	-4.00626	Down	ring finger and FYVE like domain containing E3 ubiquitin protein ligase
TMEM130	-1.57294	0.000437	0.015643	-3.72015	Down	transmembrane protein 130
KCNMB4	-1.57256	0.003907	0.048656	-3.0005	Down	potassium calcium-activated channel subfamily M regulatory beta subunit 4
LINC00924	-1.57057	0.000165	0.009593	-4.01761	Down	long intergenic non-protein coding RNA 924
EREG	-1.57034	0.000125	0.008283	-4.09939	Down	epiregulin
URAD	-1.56639	0.000704	0.020012	-3.5708	Down	ureidoimidazoline (2-oxo-4-hydroxy-4-carboxy-5-) decarboxylase
SLC22A15	-1.56367	0.000564	0.017736	-3.64079	Down	solute carrier family 22 member 15
ADRA2B	-1.56339	0.000592	0.018192	-3.62512	Down	adrenoceptor alpha 2B
CDH26	-1.56317	0.00033	0.013567	-3.80719	Down	cadherin 26
ZNF596	-1.5628	0.000227	0.01115	-3.92153	Down	zinc finger protein 596
NYAP1	-1.55572	3.9E-05	0.004533	-4.43763	Down	neuronal tyrosine phosphorylated phosphoinositide-3-kinase adaptor 1
SFRP5	-1.55537	0.000468	0.016151	-3.699	Down	secreted frizzled related protein 5
LOC101929574	-1.55461	0.001368	0.027894	-3.35602	Down	uncharacterized LOC101929574
RNF207	-1.54999	7.31E-06	0.002243	-4.90599	Down	ring finger protein 207
NRL	-1.54936	2.58E-05	0.003634	-4.55549	Down	neural retina leucine zipper
ANGPTL6	-1.54763	0.00025	0.011782	-3.89133	Down	angiopoietin like 6
ALOX15	-1.54749	0.000145	0.009003	-4.0563	Down	arachidonate 15-lipoxygenase
CHEK2	-1.54682	0.001992	0.03385	-3.23132	Down	checkpoint kinase 2
ZNF543	-1.54508	0.00303	0.042657	-3.08876	Down	zinc finger protein 543
ZNF717	-1.54439	0.000156	0.009363	-4.03319	Down	zinc finger protein 717
FASLG	-1.54428	0.001265	0.026877	-3.38181	Down	Fas ligand
LILRA3	-1.5435	1.84E-05	0.003308	-4.65038	Down	leukocyte immunoglobulin like receptor A3
NEK10	-1.54346	0.00012	0.008053	-4.11146	Down	NIMA related kinase 10
MIR1914	-1.543	0.000489	0.016519	-3.68532	Down	microRNA 1914
BATF3	-1.54097	0.000518	0.017057	-3.667	Down	basic leucine zipper ATF-like transcription factor 3
AVIL	-1.54065	0.000332	0.013573	-3.80487	Down	advillin
KLK10	-1.54034	7.07E-05	0.005956	-4.26664	Down	kallikrein related peptidase 10
ZNF689	-1.53954	0.003253	0.04422	-3.06429	Down	zinc finger protein 689
MKLN1-AS	-1.53911	0.00209	0.0347	-3.2153	Down	MKLN1 antisense RNA
LOC100128398	-1.53652	0.002092	0.034722	-3.21487	Down	uncharacterized LOC100128398
LRP1-AS	-1.53646	4.89E-05	0.005015	-4.37323	Down	LRP1 antisense RNA
NPIPA1	-1.5363	0.003113	0.043191	-3.07949	Down	nuclear pore complex interacting protein family member A1
NLRP2	-1.53625	0.00031	0.012977	-3.82661	Down	NLR family pyrin domain containing 2
MMEL1	-1.5346	0.000336	0.013596	-3.80163	Down	membrane metalloendopeptidase like 1
SPDYE5	-1.53457	0.000116	0.007921	-4.12051	Down	speedy/RINGO cell cycle regulator family member E5
CCL28	-1.5342	5.69E-05	0.005435	-4.32942	Down	C-C motif chemokine ligand 28
KLHDC1	-1.53297	0.000501	0.016732	-3.67754	Down	kelch domain containing 1
FRMPD1	-1.53279	4.28E-05	0.004701	-4.41134	Down	FERM and PDZ domain containing 1
LINC00211	-1.53271	0.00019	0.010241	-3.97414	Down	long intergenic non-protein coding RNA 211
ATP6V1G2	-1.53264	0.000799	0.021227	-3.53045	Down	ATPase H+ transporting V1 subunit G2
PRIMA1	-1.53113	0.000786	0.021056	-3.53546	Down	proline rich membrane anchor 1
NPTX1	-1.52859	0.000253	0.011808	-3.88811	Down	neuronal pentraxin 1
LOC101928107	-1.5272	0.0003	0.012783	-3.83612	Down	uncharacterized LOC101928107
GFRA3	-1.5245	8.59E-06	0.002428	-4.86161	Down	GDNF family receptor alpha 3
LOC101929595	-1.51972	1.12E-05	0.002709	-4.78841	Down	uncharacterized LOC101929595
FLJ37453	-1.51859	7.55E-06	0.002243	-4.8971	Down	uncharacterized LOC729614
MACC1	-1.51799	0.000573	0.017831	-3.6354	Down	MET transcriptional regulator MACC1
LINC00607	-1.51637	2.68E-05	0.00371	-4.5447	Down	long intergenic non-protein coding RNA 607
FAM86B1	-1.5153	0.000332	0.013573	-3.80528	Down	family with sequence similarity 86 member B1
MIR4477B	-1.51191	2.05E-05	0.00341	-4.62019	Down	microRNA 4477b
CNNM2	-1.50798	0.001668	0.03072	-3.29062	Down	cyclin and CBS domain divalent metal cation transport mediator 2
SKA1	-1.50659	0.00086	0.02226	-3.50665	Down	spindle and kinetochore associated complex subunit 1
ABRACL	-1.50603	0.001136	0.025784	-3.41701	Down	ABRA C-terminal like
LINC00954	-1.50484	0.000652	0.019135	-3.59472	Down	long intergenic non-protein coding RNA 954
ZBP1	-1.50412	0.002141	0.035221	-3.20719	Down	Z-DNA binding protein 1
ZNF528	-1.50042	0.000464	0.016097	-3.70168	Down	zinc finger protein 528
ACTG1P4	-1.50023	0.000252	0.011803	-3.88932	Down	actin gamma 1 pseudogene 4
MYO10	-1.50014	0.003103	0.043113	-3.08058	Down	myosin X

### GO and pathway enrichment of DEGs in PCOS

The top 739 DEGs were chosen to perform GO and REACTOME pathway enrichment analyses. Gene Ontology (GO) analysis identified that the DEGs were significantly enriched in BP, including the peptide metabolic process, intracellular protein transport, plasma membrane bounded cell projection organization and cell morphogenesis (Table 3). In terms of CC, DEGs were mainly enriched in organelle envelope, catalytic complex, neuron projection and cell junction were the most significantly enriched GO term (Table 3). In addition, MF demonstrated that the DEGs were enriched in the RNA binding, transcription factor binding, DNA-binding transcription factor activity, RNA polymerase II-specific and ATP binding (Table 3). REACTOME pathway enrichment analysis was used to screen the signaling pathways for differential genes. These DEGs were mainly involved in the translation, respiratory electron transport, generic transcription pathway and transmembrane transport of small molecules (Table 4).

**Table 3:** The enriched GO terms of the up and down regulated differentially expressed genes

GO ID	CATEGORY	GO Name	P Value	FDR B&H	FDR B&Y	Bonferroni	Gene Count	Gene
**Up regulated genes**								
GO:0006518	BP	peptide metabolic process	1.51E-13	2.66E-11	2.37E-10	6.37E-10	52	RPL24,RPL27,RPL30,RPL29,RPL34,RPL35A,RPL36A,RPLP0,MRPL12,MRPS12,RPS5,RPS8,ETF1,RPS10,EIF3E,RPS15,RPS15A,SRP72,RPS20,RPS21,RPS23,RPS24,RPS29,EIF6,EIF3L,MRPS21,MRPL24,MRPL20,COA3,RBM3,TRMT112,MRPL54,CPE,RPL14,RPS27L,EIF2D,DPH5,EIF1AX,ATP6AP2,ZNF706,SEC11A,MRPS24,UHMK1,EIF5,MRPS15,MRPS6,MRPS34,LSM14A,RPL35,RPL13A,MRPL9,RSL24D1
GO:0006886	BP	intracellular protein transport	6.75E-12	8.16E-10	7.28E-09	2.86E-08	66	GIPC1,SNAPIN,RPL24,TCTN3,RPL27,RPL30,RPL29,RPL34,ROMO1,RPL35A,RPL36A,RPLP0,RPS5,RPS8,RPS10,RPS15,RPS15A,PHB2,SRP72,RPS20,HMGCL,RPS21,RPS23,RPS24,RPS29,ARFIP2,TOMM5,EIF6,RAB10,ATP6V1D,SNX8,PEX19,AGFG1,PEX2,ZFAND6,RABGEF1,SNX9,RAB4A,PMM1,TOMM7,SRSF6,ANP32B,RPAIN,TNPO2,DERL2,FIS1,RPL14,APOD,ECH1,IFI27,YWHAH,TMED4,ARF5,NDUFA13,EXOC5,EIF2D,UBL5,TIMM8B,ICMT,UHMK1,DYNLL2,VPS28,RPL35,SYVN1,RPL13A,STX10
GO:0031967	CC	organelle envelope	2.36E-19	1.71E-17	1.20E-16	1.47E-16	72	ROMO1,PRELID1,MRPL12,MRPS12,PET100,BAK1,PHB2,LRRC59,HMGCL,GCHFR,COA4,NDUFA2,SLC25A26,BLOC1S1,ECSIT,SUCLG1,NDUFB2,TOMM5,NDUFC2,BANF1,MOAP1,NDUFS4,NDUFS5,PHB,BNIP3,PI4KB,FDX1,MRPS21,AGFG1,SLC25A11,TMEM97,UQCRC1,UQCRFS1,UQCRH,UQCR11,NME2,MRPL24,CERS2,MRPL20,TOMM7,COA3,COX4I1,COX5B,DTYMK,ANXA4,COX6A1,NDUFAF3,COX6B1,MRPL54,COX6C,COX7A2,COX7C,COX8A,FIS1,IFI27,MAD2L1BP,CCND2,NDUFA13,CYB5B,TMEM256,TIMM8B,AIFM1,MRPS24,MRPS15,CHCHD2,MRPS6,MRPS34,COX7A2L,UQCRQ,HSD17B10,MTDH,MRPL9
GO:1902494	CC	catalytic complex	1.31E-12	5.84E-11	4.09E-10	8.17E-10	67	PRPF31,DCAF6,ERH,SAP18,OST4,PSMB1,PSMB4,ETFB,PSMB7,PSMC5,PSMD3,PSMD10,NDUFA2,EZH1,SUCLG1,NDUFB2,NDUFC2,NDUFS4,NDUFS5,LSM3,CKS1B,TSPAN17,SLC9A3R1,UBE2E1,RBMX,MORF4L1,PEX2,AK1,PRMT1,UQCRC1,UQCRFS1,UQCRH,TAF7,ANAPC5,TADA3,ANAPC13,RBX1,CUL4A,POLR3GL,RBBP7,PIGS,POLR2J,POLR2K,DERL2,BCCIP,POLE4,NAA38,CCNC,CCND2,RMRP,NDUFA13,POLR1D,GTF2A2,ORMDL1,SEC11A,SF3B6,DYNLL2,SMARCD2,UQCRQ,HSD17B10,KLHL12,SYVN1,CDC27,PSMD14,SNRPD2,RPPH1,KRTCAP2
GO:0003723	MF	RNA binding	1.66E-10	4.49E-08	3.30E-07	1.45E-07	76	RPL24,PRPF31,RPL27,RPL30,RPL29,RPL34,RPL35A,RPL36A,ERH,RPLP0,MRPL12,MRPS12,SAP18,RSL1D1,RPS5,RPS8,ETF1,RPS10,EIF3E,RPS15,RPS15A,SRP72,RPS20,LRRC59,RPS21,RPS23,RPS24,SUCLG1,EIF6,LSM3,RBMX,EIF3L,MRPS21,NQO1,AGFG1,LSM4,PRMT1,SLC25A11,TBCA,MRPL20,NUDT16,SRSF6,S100A16,GLRX3,RBBP7,RBM3,MRPL54,NELFB,BCCIP,RDX,RPL14,C7orf50,XRCC5,SERPINH1,RPS27L,EIF2D,GTF3A,FAM32A,EIF1AX,SBDS,SF3B6,MRPS24,UHMK1,EIF5,MRPS15,SLTM,MRPS6,LSM14A,HSD17B10,RPL35,RPL13A,SNRPD2,MTDH,MRPL9,SF3B5,RSL24D1
GO:0008134	MF	transcription factor binding	1.05E-02	1.83E-01	1.00E+00	1.00E+00	23	NAB1,PHB2,PSMC5,FAM89B,NBN,PSMD10,MDFI,TAF7,SETD3,TADA3,RBX1,WIPI1,ANXA4,TEAD3,IFI27,YWHAH,GTF2A2,MED4,ICMT,CHCHD2,HSD17B10,MTDH,TRIB1
**Down regulated genes**								
GO:0120036	BP	plasma membrane bounded cell projection organization	7.28E-05	2.19E-02	1.94E-01	2.91E-01	49	EPHB4,STK36,MAK,SPAST,ZFYVE27,TTLL3,MYO7A,MYO10,SDCCAG8,SPTB,SARM1,RAP1GAP2,NPHP4,GFRA3,AMIGO1,HECW2,GLI2,GLI3,SYNE2,WRAP73,NGFR,NTN1,NLGN2,FGFR3,PLD1,CEP126,NYAP1,DNAH5,UBXN10,PLXNB3,SEMA6B,FAT4,NPTX1,NEDD4L,STXBP5,FBF1,LAMA5,RFFL,ODF2,FAS,RFX3,ATL1,TRAK1,WDR19,FEZ1,AVIL,CEP135,IFT122,EPHA4
GO:0000902	BP	cell morphogenesis	3.10E-03	1.93E-01	1.00E+00	1.00E+00	31	EPHB4,CDH26,SPAST,ZFYVE27,MYO7A,MYO10,CDHR3,SPTB,SARM1,GFRA3,AMIGO1,HECW2,GLI2,GLI3,NGFR,NTN1,FGFR3,NYAP1,PLXNB3,SEMA6B,NPTX1,NEDD4L,STXBP5,LAMA5,CDH19,ATL1,TRAK1,WDR19,FEZ1,C8orf37,EPHA4
GO:0043005	CC	neuron projection	3.65E-05	7.26E-03	5.00E-02	2.00E-02	46	EPHB4,MAK,PDE4B,SNPH,SPAST,ZFYVE27,MYO7A,MYO10,ESR1,SDCCAG8,SARM1,RAP1GAP2,N4BP3,NPHP4,SNAP47,TIAM2,AMIGO1,KCNC1,PTPRN2,SYNJ2,NGFR,NLGN2,BSN,NPTX1,CALCR,PLXDC1,STXBP5,ERO1A,GRK4,AAK1,APBA1,KLC2,VTI1A,FAS,ATL1,TRAK1,WDR19,SHISA9,FEZ1,KCNQ4,AVIL,UNC13C,IFT122,CTNND1,EPHA4,GABRA4
GO:0030054	CC	cell junction	3.04E-03	8.34E-02	5.74E-01	1.00E+00	34	CDH26,SNPH,CDHR3,SDCCAG8,SARM1,OBSCN,NPHP4,ABCB4,ARHGAP22,RASIP1,PTPRN2,SYNE2,LPXN,NGFR,NLGN2,FGFR3,BSN,FHL3,CDC42BPA,PLXDC1,STXBP5,FBF1,LCK,CDH19,CLDN1,PRIMA1,SHISA9,LMO7,CTNNA3,UNC13C,C8orf37,CTNND1,EPHA4,GABRA4
GO:0000981	MF	DNA-binding transcription factor activity, RNA polymerase II-specific	9.75E-04	3.67E-01	1.00E+00	7.35E-01	43	ZNF717,ZXDC,HIVEP1,ERG,ZNF154,ESR1,ZSCAN25,ARNTL2,ZNF836,ZNF469,ZNF547,GLI2,GLI3,ZNF347,ZNF778,BATF3,TET3,ZNF687,ZNF114,MACC1,ZNF555,ZNF493,FOXP2,NRL,ZNF596,ZNF689,ZNF300,ZKSCAN2,RFX3,ZNF496,IKZF4,RLF,KAT7,DMRT2,ZNF334,MTF1,ZKSCAN3,ZNF559-ZNF177,ZNF69,ZNF528,ZNF543,GLIS1,ZNF124
GO:0005524	MF	ATP binding	2.83E-02	4.14E-01	1.00E+00	1.00E+00	34	EPHB4,PRPS2,STK36,MAK,CNNM2,SPAST,TTLL3,MYO7A,MYO10,NEK10,OBSCN,PFAS,TOR4A,ABCB4,FGFR3,XYLB,DNAH5,DNHD1,ATP13A4,DNAH3,CDC42BPA,ATP13A1,GRK4,MAP 3K14,AAK1,ABCC5,LCK,NLRP2,NLRP6,TRIB3,CHEK2,ERCC6L2,MAST3,EPHA4

*BP* Biological Process, *CC* Cellular Component and *MF* Molecular Functions

**Table 4:** The enriched pathway terms of the up and down regulated differentially expressed genes

Pathway ID	Pathway Name	P-value	FDR B&H	FDR B&Y	Bonferroni	Gene Count	Geness
Up regulated genes							
1268678	Translation	8.83E-20	4.54E-17	3.26E-16	6.54E-17	30	RPL24,RPL27,RPL30,RPL29,RPL34,RPL35A,RPL36A,RPLP0,RPS5,RPS8,ETF1,RPS10,EIF3E,RPS15,RPS15A,SRP72,RPS20,RPS21,RPS23,RPS24,RPS29,EIF3L,TRMT112,RPL14,RPS27L,EIF1AX,SEC11A,EIF5,RPL35,RPL13A
1270128	Respiratory electron transport	2.39E-16	1.27E-14	9.10E-14	1.77E-13	22	ETFB,NDUFA2,ECSIT,NDUFB2,NDUFC2,NDUFS4,NDUFS5,UQCRC1,UQCRFS1,UQCRH,UQCR11,COX4I1,COX5B,COX6A1,NDUFAF3,COX6B1,COX6C,COX7C,COX8A,NDUFA13,COX7A2L,UQCRQ
1268677	Metabolism of proteins	6.29E-10	1.46E-08	1.05E-07	4.66E-07	72	B4GALT2,RPL24,RPL27,RPL30,RPL29,UAP1,RPL34,RPL35A,RPL36A,RPLP0,RPS5,PSMB1,RPS8,ETF1,RPS10,PSMB4,ETFB,EIF3E,PSMB7,RPS15,RPS15A,PSMC5,SRP72,PSMD3,RPS20,PFDN1,RPS21,RPS23,PFDN5,PSMD10,RPS24,COA4,RPS29,TOMM5,GFPT1,NEDD8,RAB10,SAA1,UBE2E1,UCHL1,EIF3L,TADA3,RAB4A,TBCA,PMM1,TOMM7,WIPI1,TRMT112,TNIP2,PIGS,AMDHD2,USP24,DERL2,CPE,RPL14,RPS27L,ARF5,EXOC5,TIMM8B,DPH5,ICMT,EIF1AX,ATP6AP2,SEC11A,DYNLL2,EIF5,CHCHD2,SUMO3,RPL35,SYVN1,RPL13A,PSMD14
1269649	Gene Expression	1.72E-09	3.85E-08	2.77E-07	1.27E-06	77	CDKN2B,RPL24,PRPF31,RPL27,RPL30,RPL29,RPL34,RPL35A,RPL36A,PRELID1,TMEM219,RPLP0,SAP18,RPS5,PSMB1,RPS8,ETF1,RPS10,PSMB4,EIF3E,PSMB7,RPS15,RPS15A,PSMC5,NBN,SRP72,PSMD3,RPS20,RPS21,RPS23,PSMD10,RPS24,RPS29,LSM3,RBMX,EIF3L,NABP2,LSM4,PRMT1,TAF7,SRSF6,POLR3GL,RBBP7,COX4I1,TRMT112,COX5B,COX6A1,COX6B1,POLR2J,POLR2K,NELFB,COX6C,COX7C,COX8A,RPL14,TEAD3,SKIL,YWHAH,RPS27L,CCNC,POLR1D,GTF2A2,MED4,GTF3A,LAMTOR4,EIF1AX,ZNF706,SEC11A,SF3B6,EIF5,COX7A2L,HSD17B10,RPL35,RPL13A,PSMD14,SNRPD2,SF3B5
1269852	Autodegradation of Cdh1 by Cdh1:APC/C	1.22E-06	2.58E-05	1.86E-04	9.04E-04	10	PSMB1,PSMB4,PSMB7,PSMC5,PSMD3,PSMD10,UBE2E1,ANAPC5,CDC27,PSMD14
1268843	Mitochondrial translation initiation	2.55E-06	4.96E-05	3.57E-04	1.89E-03	11	MRPL12,MRPS12,MRPS21,MRPL24,MRPL20,MRPL54,MRPS24,MRPS15,MRPS6,MRPS34,MRPL9
**Down regulated genes**							
1269650	Generic Transcription Pathway	1.96E-04	1.10E-01	7.63E-01	1.10E-01	29	ZNF717,ZNF486,ZNF154,ESR1,ZSCAN25,MEN1,ZNF547,BANP,ZNF347,ZNF778,TAF4B,ZNF114,ZNF573,ZNF555,ZNF493,NEDD4L,ZNF596,ZNF689,ZNF300,RFFL,FAS,ZNF496,ZNF334,ZKSCAN3,CHEK2,ZNF528,ZNF543,TOP3A,ZNF124
1269903	Transmembrane transport of small molecules	2.80E-02	7.66E-01	1.00E+00	1.00E+00	18	ATP6V1G2,SLC22A15,SLCO4A1,ASIC3,ABCB4,SLC24A4,SLC44A5,SCNN1D,SLC9A7,ATP13A4,NEDD4L,ATP13A1,ABCC5,SLC1A7,SLC5A4,SLC30A7,NDC1,GABRA4
1268846	Cilium Assembly	3.71E-02	7.66E-01	1.00E+00	1.00E+00	7	SDCCAG8,NPHP4,FBF1,ODF2,WDR19,CEP135,IFT122
1269957	Metabolism of carbohydrates	1.11E-01	7.66E-01	1.00E+00	1.00E+00	8	PRPS2,CHST14,XYLB,MGAM,ABCC5,SLC5A4,NDC1,SLC25A13
1269443	Signalling by NGF	1.63E-01	7.66E-01	1.00E+00	1.00E+00	11	EREG,SPTB,OBSCN,KBTBD7,GFRA3,TIAM2,NGFR,FGFR3,BCL2L11,LCK,TRIB3
1269171	Adaptive Immune System	1.86E-01	7.66E-01	1.00E+00	1.00E+00	17	EREG,LRSAM1,HLA-H,RAP1GAP2,KBTBD7,SLAMF6,HECW2,FGFR3,KIR2DL4,TREML1,LILRA3,NEDD4L,MAP 3K14,KLC2,LCK,TRIB3,LMO7

### PPI networks construction and module Analysis

**Fig. 3:**
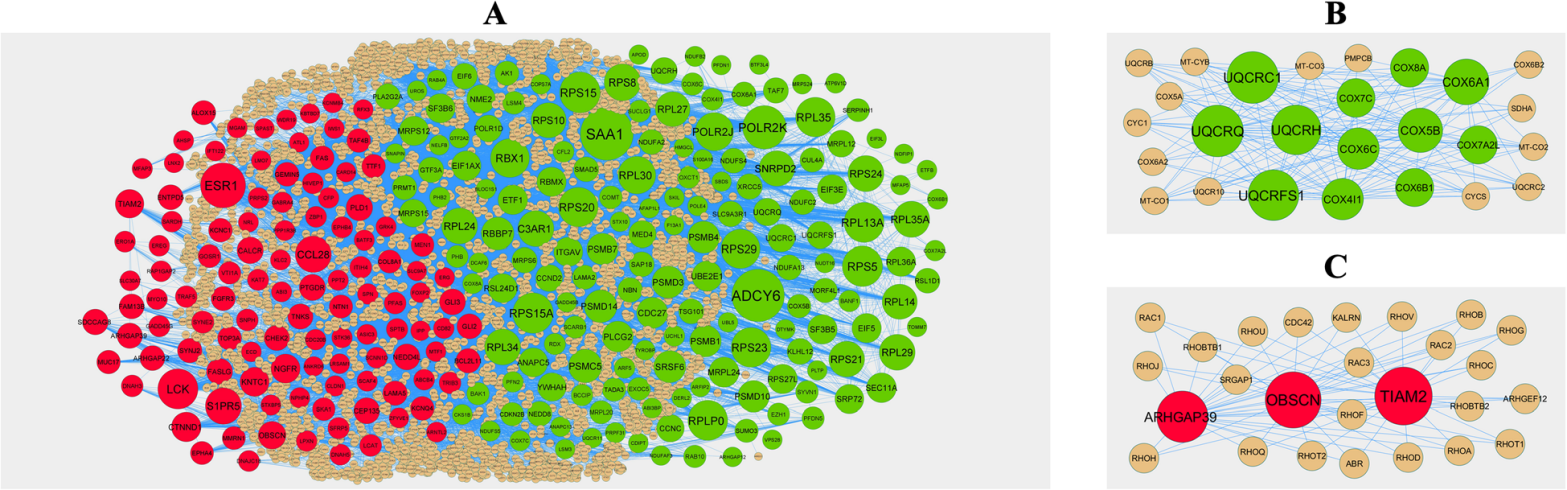
PPI network and the most significant modules of DEGs. **a** The PPI network of DEGs was constructed using Cytoscape. **b** The most significant module was obtained from PPI network with 26 nodes and 160 edges for up regulated genes. **c** The most significant module was obtained from PPI network with 26 nodes and 71 edges for up regulated genes. Up regulated genes are marked in green; down regulated genes are marked in red

Following the analysis based on the PPI networks, 4141 nodes and 14853 edges were identified in Cytoscape (Fig. 3a). The genes with higher scores were the hub genes, as the genes of node degree, betweenness centrality, stress centrality, closeness centrality may be linked with PCOS. The top 10 hub genes were SAA1, ADCY6, POLR2K, RPS15, RPS15A, ESR1, LCK, S1PR5, CCL28 and CTNND1 and are listed in Table 5. Enrichment analysis demonstrated that module 1 (Fig. 3b) and module 2 (Fig. 3c) might be associated with respiratory electron transport, organelle envelope, catalytic complex, gene expression, signaling by NGF and neuron projection.

**Table 5:** Topology table for up and down regulated genes

Regulation	Node	Degree	Betweenness	Stress	Closeness
Up	SAA1	315	0.10107	69407430	0.321603
Up	ADCY6	312	0.072777	44726506	0.302897
Up	POLR2K	236	0.037471	28379862	0.302013
Up	RPS15	212	0.008152	26431684	0.308863
Up	RPS15A	211	0.007809	26602340	0.309741
Up	RPS5	209	0.007859	26492428	0.309463
Up	RPL13A	207	0.007846	22124842	0.309533
Up	RPS23	205	0.005989	23951504	0.308794
Up	RPLP0	205	0.00818	22450450	0.310438
Up	RPL35	203	0.006165	21837168	0.309417
Up	RPS20	202	0.005559	24073032	0.308564
Up	RPS29	198	0.004498	20672292	0.308426
Up	RBX1	197	0.044386	26711280	0.318217
Up	POLR2J	192	0.020679	20252592	0.293659
Up	RPL30	187	0.006891	19563996	0.308265
Up	RPS8	177	0.004042	18164736	0.30678
Up	RPL35A	174	0.003759	16445648	0.30783
Up	RPS24	174	0.003192	15939464	0.304838
Up	RPL24	173	0.003533	16772430	0.306803
Up	C3AR1	172	0.003177	5121210	0.274955
Up	RPL34	172	0.004039	16061864	0.30719
Up	RPL29	171	0.00494	18453558	0.307647
Up	RPS21	170	0.002685	15196960	0.304636
Up	SNRPD2	168	0.03814	33998368	0.309486
Up	RPS10	164	0.002949	14807414	0.306055
Up	RPL14	163	0.00328	15070824	0.306826
Up	RPL27	160	0.002277	14404254	0.305716
Up	PSMC5	145	0.016771	20051716	0.327066
Up	PSMD14	138	0.008797	20693344	0.312311
Up	RBBP7	136	0.039699	19307334	0.314733
Up	PSMD3	132	0.006138	19507196	0.312264
Up	SRSF6	130	0.01551	18603044	0.305242
Up	PSMB4	128	0.006024	19460760	0.31217
Up	PSMB1	126	0.005935	19159108	0.312736
Up	PSMD10	125	0.006564	19293068	0.313162
Up	PSMB7	121	0.004879	18406276	0.311911
Up	CDC27	121	0.014082	17205180	0.314709
Up	PLCG2	119	0.031441	10514426	0.284595
Up	UBE2E1	111	0.01698	18575766	0.315597
Up	MRPL24	111	0.004006	3985902	0.27419
Up	SEC11A	107	0.005316	8457436	0.302168
Up	EIF3E	105	0.00271	6755804	0.298099
Up	EIF1AX	105	0.00175	6248608	0.298099
Up	ETF1	104	0.003209	7817310	0.300348
Up	SF3B5	104	0.003133	6307886	0.271298
Up	EIF5	103	0.002157	5951468	0.298056
Up	MRPS12	103	0.001566	3803642	0.27381
Up	RPS27L	103	0.003987	8037762	0.3043
Up	RBMX	102	0.006533	8629778	0.302543
Up	SF3B6	101	0.007747	10034416	0.30272
Up	RPL36A	101	0.001189	3109720	0.271102
Up	RSL24D1	100	0.010421	6175826	0.267684
Up	ANAPC5	100	0.005802	14180882	0.312854
Up	ITGAV	98	0.047765	36204220	0.313518
Up	SRP72	98	0.001539	6042806	0.299696
Up	NME2	94	0.034644	15645818	0.312029
Up	YWHAH	93	0.030402	22230232	0.316369
Up	MRPS15	86	0.001926	2385992	0.260394
Up	UQCRQ	83	0.003421	1615130	0.240866
Up	CCNC	83	0.010303	8511008	0.281231
Up	UQCRC1	82	0.02328	14231790	0.289875
Up	MED4	80	0.018252	17775072	0.301683
Up	UQCRFS1	78	0.001699	1157234	0.244233
Up	CCND2	77	0.023553	14830028	0.314399
Up	MRPL12	77	0.002782	2194820	0.259789
Up	POLR1D	75	0.0061	4678140	0.274081
Up	UQCRH	72	0.001221	959168	0.244147
Up	KLHL12	68	0.003828	3608276	0.303719
Up	SLC9A3R1	63	0.021981	10906810	0.303987
Up	XRCC5	63	0.016826	12450448	0.298895
Up	EIF6	62	0.005109	3166876	0.251672
Up	GTF3A	61	0.016181	6249890	0.308426
Up	MRPS6	60	6.26E-04	932952	0.249563
Up	PLA2G2A	59	0.016536	3353206	0.267442
Up	NDUFC2	58	0.009137	6243750	0.288562
Up	TAF7	58	0.008456	6200048	0.248053
Up	NDUFA13	58	0.010438	6851160	0.289086
Up	TSG101	57	0.016521	11031600	0.306145
Up	SAP18	57	0.012307	5315482	0.302256
Up	NDUFS4	56	0.001242	656204	0.239889
Up	SMAD5	56	0.020424	14462992	0.296201
Up	RSL1D1	56	0.011475	16770568	0.229134
Up	NDUFA2	54	7.42E-04	556554	0.239861
Up	NBN	54	0.015436	11207188	0.30323
Up	NEDD8	53	0.011483	3600034	0.320831
Up	PRMT1	52	0.012592	15575898	0.301332
Up	MORF4L1	51	0.010667	4279006	0.296795
Up	CUL4A	50	0.007675	2678820	0.299544
Up	LAMA2	49	0.007709	8570206	0.278844
Up	COX6A1	48	0.001525	801088	0.240334
Up	AK1	46	0.003174	1615960	0.250894
Up	COX5B	42	0.001215	595708	0.239861
Up	TADA3	40	0.010349	2368830	0.281307
Up	SUMO3	40	0.011044	5466788	0.300348
Up	CDKN2B	36	0.006521	2033654	0.280983
Up	SERPINH1	36	0.01212	7196482	0.29909
Up	MRPL20	34	0.002069	1471944	0.293201
Up	COMT	34	0.015004	4152910	0.286109
Up	LSM4	34	0.007332	2056240	0.254456
Up	EXOC5	33	0.00443	1785404	0.263041
Up	SYVN1	32	0.012143	5434366	0.291078
Up	BAK1	31	0.006508	1183542	0.267425
Up	RAB10	30	0.008921	6109230	0.296328
Up	PFDN5	29	0.010966	3969486	0.290567
Up	SCARB1	28	0.010079	5204710	0.299544
Up	COX6C	27	4.36E-04	219006	0.232011
Up	COX4I1	26	6.32E-04	186792	0.232493
Up	EZH1	26	0.001591	543666	0.245377
Up	SUCLG1	26	0.008369	8540110	0.200727
Up	OXCT1	25	0.007475	2818178	0.199778
Up	PHB	24	0.005122	2747910	0.293388
Up	BANF1	20	0.005143	1975740	0.295651
Up	BCCIP	20	0.003654	1838164	0.269444
Up	RDX	18	0.004798	4656272	0.29563
Up	CFL2	15	0.005519	1602836	0.286624
Up	ARF5	12	0.004972	1234824	0.284419
Up	UCHL1	12	0.001629	954606	0.29618
Up	PFN2	11	0.003745	1598036	0.28734
Up	NDUFB2	8	0	0	0.225404
Up	COX6B1	8	0	0	0.225073
Up	COX7A2L	8	0	0	0.225073
Up	COX7C	8	0	0	0.225073
Up	PRPF31	8	1.73E-04	119394	0.213127
Up	COX8A	8	0	0	0.225073
Up	NDUFS5	8	0	0	0.225404
Up	PHB2	8	0.001363	712242	0.308449
Up	ETFB	4	0	0	0.224963
Up	NELFB	3	2.95E-04	570978	0.27419
Up	CKS1B	3	6.28E-06	1544	0.254284
Up	ANAPC13	3	0	0	0.245741
Up	GTF2A2	3	0	0	0.236342
Up	POLE4	3	2.39E-04	114402	0.254863
Up	TYROBP	2	0	0	0.248948
Up	DCAF6	2	0	0	0.241724
Up	SKIL	2	0	0	0.239764
Up	S100A16	2	0	0	0.172622
Up	HMGCL	2	0	0	0.172622
Up	ARHGAP12	2	7.30E-07	10130	0.210056
Up	STX10	2	0	0	0.222748
Up	UQCR11	2	0	0	0.194129
Up	GADD45B	1	0	0	0.188748
Up	PFDN1	1	0	0	0.225159
Up	CDIPT	1	0	0	0.221556
Up	TOMM7	1	0	0	0.211009
Up	MRPS24	1	0	0	0.206607
Up	SBDS	1	0	0	0.201078
Up	COPS7A	1	0	0	0.230512
Up	DERL2	1	0	0	0.225466
Up	DTYMK	1	0	0	0.237835
Up	NDFIP1	1	0	0	0.237726
Up	BTF3L4	1	0	0	0.213292
Up	AFAP1L1	1	0	0	0.195053
Up	ATP6V1D	1	0	0	0.235992
Up	LSM3	1	0	0	0.202852
Up	ARFIP2	1	0	0	0.22145
Up	F13A1	1	0	0	0.201558
Up	ABI3BP	1	0	0	0.18668
Up	NDUFAF3	1	0	0	0.193485
Up	APOD	1	0	0	0.201401
Up	UBL5	1	0	0	0.242915
Up	MFAP5	1	0	0	0.160727
Up	RAB4A	1	0	0	0.234402
Up	SNAPIN	1	0	0	0.232493
Up	UROS	1	0	0	0.212264
Up	PLTP	1	0	0	0.201401
Up	VPS28	1	0	0	0.234402
Up	EIF3L	1	0	0	0.229656
Up	NUDT16	1	0	0	0.237835
Up	BLOC1S1	1	0	0	0.256919
Down	ESR1	250	0.13227	1.07E+08	0.34572
Down	LCK	209	0.099202	95979964	0.328259
Down	S1PR5	174	0.006652	8092966	0.28169
Down	CCL28	174	0.003314	5208440	0.274992
Down	CTNND1	106	0.042469	42968374	0.310275
Down	KNTC1	101	0.02909	80024314	0.25565
Down	NGFR	100	0.036602	63182380	0.318388
Down	TIAM2	95	0.021154	62011932	0.265317
Down	OBSCN	86	0.010365	36096562	0.247534
Down	PLD1	73	0.033394	17246708	0.30421
Down	CALCR	71	0.002592	2172836	0.25466
Down	PTGDR	70	0.002067	1966748	0.255524
Down	FASLG	69	0.019813	7248566	0.301244
Down	NEDD4L	68	0.023391	18526624	0.311841
Down	GLI3	68	0.017183	7308126	0.2879
Down	CEP135	65	0.013666	21907982	0.267857
Down	SDCCAG8	65	0.013666	21907982	0.267857
Down	ARHGAP39	61	0.006186	28702190	0.252655
Down	TNKS	60	0.004206	3754360	0.297842
Down	CHEK2	60	0.016237	7288936	0.307601
Down	GLI2	58	0.012509	5073890	0.282922
Down	ENTPD5	57	0.005964	2743528	0.250015
Down	FAM13B	56	0.004818	23506158	0.251122
Down	ARHGAP22	56	0.004818	23506158	0.251122
Down	LAMA5	52	0.00723	12112106	0.258896
Down	SYNJ2	52	0.010084	13175450	0.253879
Down	BCL2L11	52	0.016836	10636772	0.313969
Down	ALOX15	49	0.008753	5227944	0.244161
Down	FGFR3	49	0.011331	25238840	0.255587
Down	GEMIN5	47	0.001525	2623902	0.254973
Down	FAS	47	0.009545	4674560	0.293347
Down	PDE4B	46	0.005717	3857072	0.252024
Down	KCNC1	45	0.011352	17732982	0.215233
Down	TAF4B	45	0.004747	2685668	0.266101
Down	TOP3A	45	0.011616	46078906	0.231272
Down	TTF1	43	0.001327	799808	0.270889
Down	KCNQ4	42	0.027528	31515762	0.226874
Down	MUC17	42	0.019711	25257640	0.157086
Down	NTN1	40	0.012007	10110832	0.266152
Down	COL8A1	38	0.013121	17046748	0.212558
Down	MMRN1	38	0.011827	11815884	0.252424
Down	EPHA4	38	0.003668	1364992	0.273358
Down	MEN1	35	0.006804	6211974	0.272297
Down	GOSR1	34	0.008526	3939382	0.285714
Down	SKA1	32	0.002897	9913724	0.243129
Down	VTI1A	31	0.007925	4181998	0.286386
Down	SYNE2	30	0.007519	9249888	0.228212
Down	ITIH4	26	0.007262	1264158	0.272297
Down	LCAT	26	0.005782	971078	0.252178
Down	TRIB3	24	0.002492	6242350	0.248261
Down	TRAF5	24	0.00684	5476244	0.212395
Down	DNAH3	24	0.007222	8565654	0.215524
Down	PFAS	24	0.014988	8957870	0.288321
Down	SNPH	24	0.00801	15545944	0.224536
Down	GADD45G	20	0.003326	13947820	0.23265
Down	SFRP5	20	0.006774	5015824	0.211927
Down	SPTB	20	0.0063	11636704	0.233978
Down	KAT7	18	0.003392	3121342	0.235683
Down	EPHB4	18	0.001434	905566	0.260034
Down	DNAH5	17	0.002849	3119232	0.205745
Down	HIVEP1	17	0.005211	11367638	0.232859
Down	DNAJC18	16	0.004758	4513144	0.228489
Down	SCNN1D	16	0.001683	759732	0.291057
Down	ZBP1	16	0.0032	6672792	0.233555
Down	RAP1GAP2	15	0.003133	3581154	0.225872
Down	SARDH	15	0.005309	5233702	0.183926
Down	PRPS2	15	0.006081	1607396	0.285281
Down	MYO10	15	0.004221	1310316	0.235723
Down	ABCB4	14	5.59E-04	1308566	0.227248
Down	SPN	13	0.003074	2890384	0.244522
Down	ERO1A	13	0.004612	1425588	0.287161
Down	LPXN	13	0.002728	4156340	0.242304
Down	CD82	12	0.001605	269660	0.279107
Down	CFP	12	0.004063	2388686	0.220588
Down	CDC20B	12	7.24E-04	204270	0.243429
Down	EREG	11	0.00292	4060738	0.221568
Down	GABRA4	11	0.004826	844010	0.188362
Down	STK36	10	5.16E-06	1410	0.231582
Down	CLDN1	10	0.002949	1746194	0.196311
Down	SCAF4	10	0.003013	2202372	0.198039
Down	MFAP3	9	0.003861	3290616	0.191498
Down	PPT2	9	0.003141	1767236	0.210548
Down	NPHP4	9	0.003191	1398268	0.215199
Down	ABI3	8	0.001847	2267982	0.229515
Down	BATF3	8	6.07E-04	393104	0.226291
Down	KLC2	8	0.00245	1875364	0.222485
Down	GRK4	8	5.59E-04	417560	0.222533
Down	ARNTL2	7	0.001968	1639758	0.209684
Down	PPP1R3B	7	0.002897	1494078	0.211732
Down	IFT122	7	0.001127	614274	0.187492
Down	AHSP	7	0.002414	2877402	0.186226
Down	ASIC3	7	0.001895	501964	0.235361
Down	WDR19	7	0.001287	982818	0.187492
Down	MGAM	7	0.002455	406020	0.181388
Down	STXBP5	6	0.00108	832104	0.196993
Down	SLC30A7	6	0.002041	1162028	0.211776
Down	NRL	6	0.001496	1056416	0.226267
Down	KCNMB4	5	0.001932	644596	0.189223
Down	SPAST	5	0.001932	3015012	0.17458
Down	MTF1	5	8.87E-04	1194192	0.226862
Down	FOXP2	5	0.001059	361276	0.219804
Down	IWS1	4	4.91E-04	486888	0.21999
Down	LRSAM1	4	4.66E-04	327264	0.292456
Down	ECD	4	3.00E-08	6	0.246517
Down	RFX3	3	9.66E-04	260498	0.235696
Down	IPP	3	4.83E-04	255296	0.199412
Down	ERG	3	0.001157	1993680	0.288401
Down	SLC9A7	2	4.83E-04	102030	0.189205
Down	KBTBD7	2	4.83E-04	116108	0.284029
Down	CARD14	2	2.67E-05	3564	0.219501
Down	ANKRD6	2	3.60E-07	366	0.212744
Down	ZFYVE1	2	4.83E-04	123952	0.234428
Down	LNX2	1	0	0	0.215132
Down	ATL1	1	0	0	0.148637
Down	LMO7	1	0	0	0.195467

### Construction of miRNA - target regulatory network

**Fig. 4:**
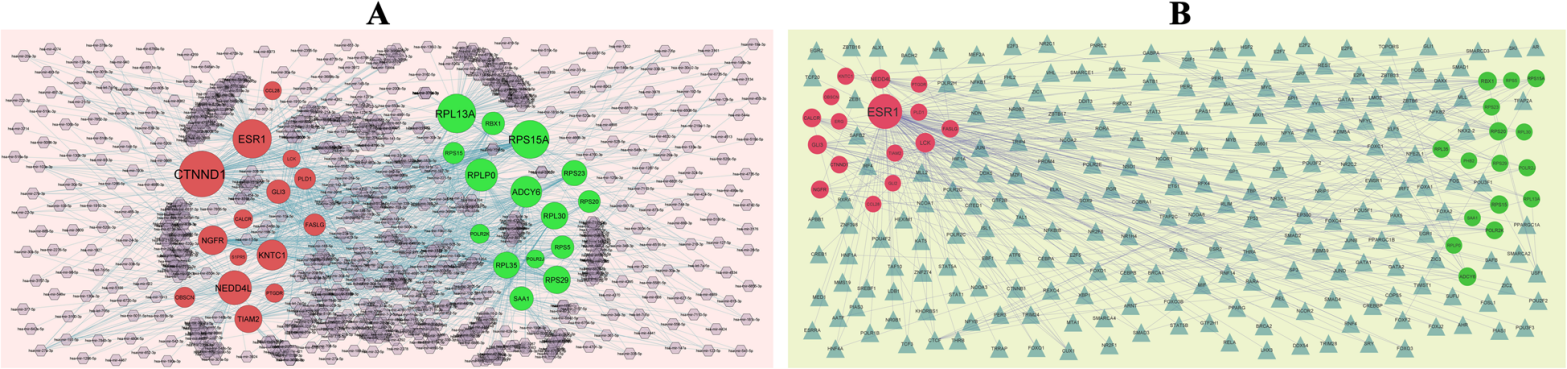
**a** Target gene - miRNA regulatory network between target genes and miRNAs. **b** Target gene - TF regulatory network between target genes and TFs. Up regulated genes are marked in green; down regulated genes are marked in red; The purple color diamond nodes represent the key miRNAs; the blue color triangle nodes represent the key TFs.

After combining the results of miRNA-target genes with the interactive network of miRNAs, 281 hub genes were selected and 2138 were miRNAs. The genes and miRNAs are shown in Fig. 4a. Specifically, 97 miRNAs (ex, hsa-mir-8067) that regulate RPL13A, 95 miRNAs (ex, hsa-mir-4518) that regulate RPS15A, 71 miRNAs (ex, hsa-mir-3685) that regulate RPLP0, 65 miRNAs (ex, hsa-mir-1202) that regulates ADCY6, 48 miRNAs (ex, hsa-mir-4461) that regulate RPS29, 129 miRNAs (ex, hsa-mir-8082) that regulate CTNND1, 98 miRNAs (ex, hsa-mir-4422) that regulate ESR1, 76 miRNAs (ex, hsa-mir-548am-5p) that regulate NEDD4L, 62 miRNAs (ex, hsa-mir-6886-3p) that regulate KNTC1 and 56 miRNAs (ex, hsa-mir-9500) that regulate NGFR were detected (Table 6).

### Construction of TF - target regulatory network

After combining the results of TF-target genes with the interactive network of TFs, 455 hub genes were selected and 274 were TFs. The genes and TFs are shown in Fig. 4b. Specifically, 15 TFs (ex, PER3) that regulate RBX1, 13 TFs (ex, CTCF) that regulate RPS15, 12 TFs (ex, E2F7) that regulate RPS20, 11 TFs (ex, LMO2) that regulate ADCY6, 9 TFs (ex, POLR2H) that regulate POLR2K, 122 TFs (ex, NCOA2) that regulate ESR1, 21 miRNAs (ex, EBF1) that regulate LCK, 18 TFs (ex, SMAD2) that regulate GLI3, 17 TFs (ex, JUND) that regulate NEDD4L, and 15 TFs (ex, FOXO3) that regulate CALCR were detected (Table 6).

### Receiver operating characteristic (ROC) curve analysis

**Fig. 5:**
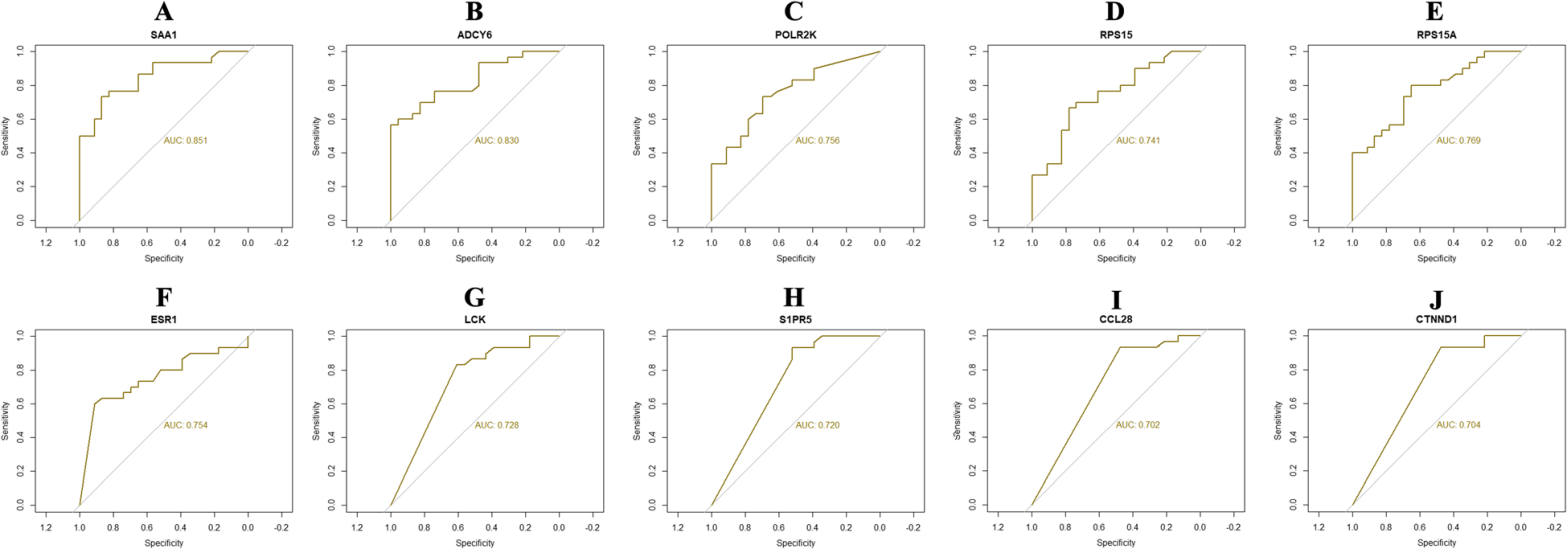
ROC curve validated the sensitivity, specificity of hub genes as a predictive biomarker for PCOS prognosis. **a** SAA1, **b** ADCY6, **c** POLR2K, **d** RPS15, **e** RPS15A, **f** ESR1, **g** LCK, **h** S1PR5, **i** CCL28, **j** CTNND1

Moreover, ROC curve analysis using “pROC” packages was performed to calculate the capacity of ten hub genes to distinguish PCOS from normal control. SAA1, ADCY6, POLR2K, RPS15, RPS15A, CTNND1, ESR1, NEDD4L, KNTC1 and NGFR all exhibited excellent diagnostic efficiency (AUC > 0.7) (Fig. 5).

### Validation of the expression levels of hub genes by RT-PCR

**Fig. 6:**
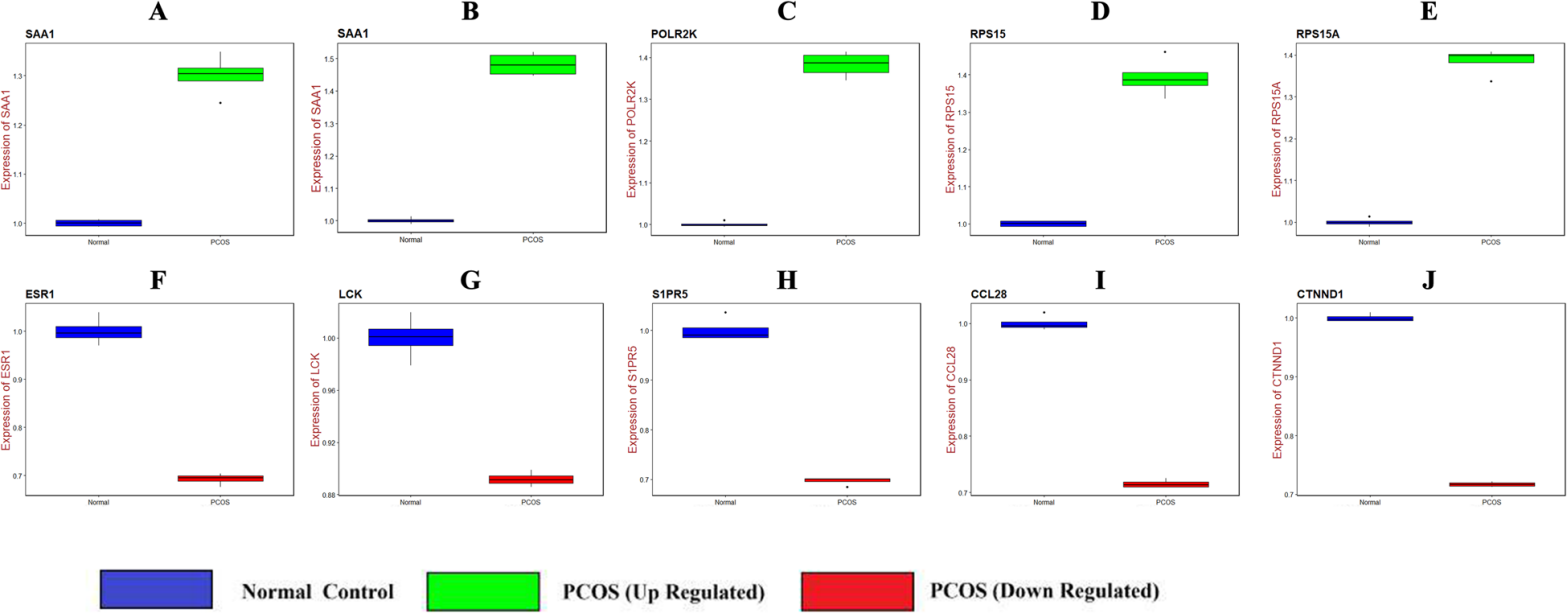
Validation of hub genes by RT- PCR. **a** SAA1, **b** ADCY6, **c** POLR2K, **d** RPS15, **e** RPS15A, **f** ESR1, **g** LCK, **h** S1PR5, **i** CCL28, **j** CTNND1

Aiming to further verify the expression patterns of selected hub genes, real-time PCR, which allows quantitative analysis of hub gene expression, was applied. The results showed that the relative expression levels of 10 hub genes including SAA1, ADCY6, POLR2K, RPS15, RPS15A, CTNND1, ESR1, NEDD4L, KNTC1 and NGFR were consistent with the expression profiling by high throughput sequencing (Fig. 6).

### Molecular docking studies

**Fig. 7:**
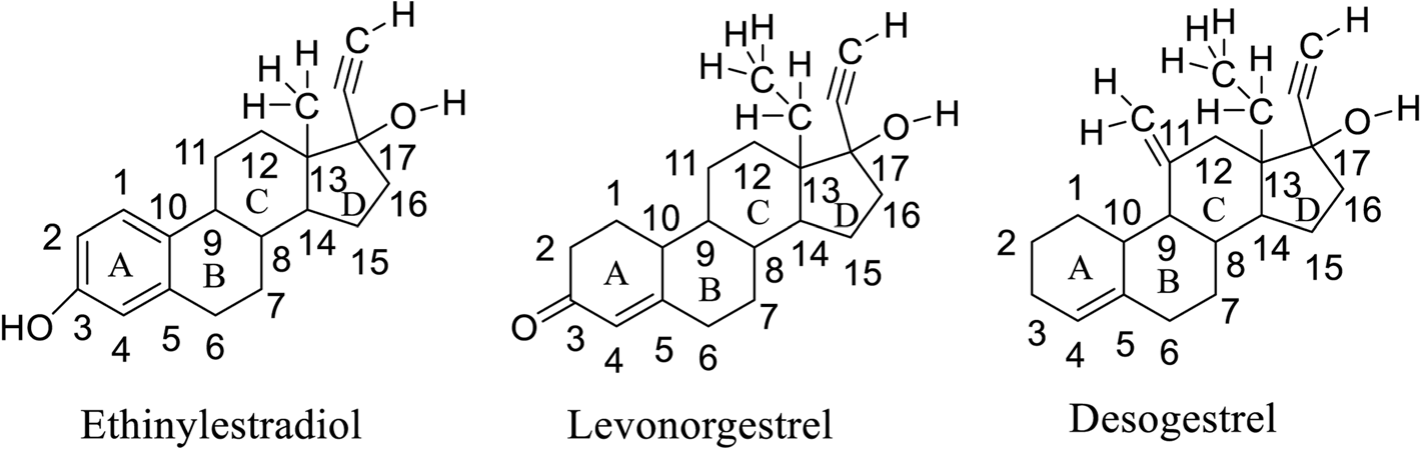
Structures of Designed Molecules

**Fig. 8:**
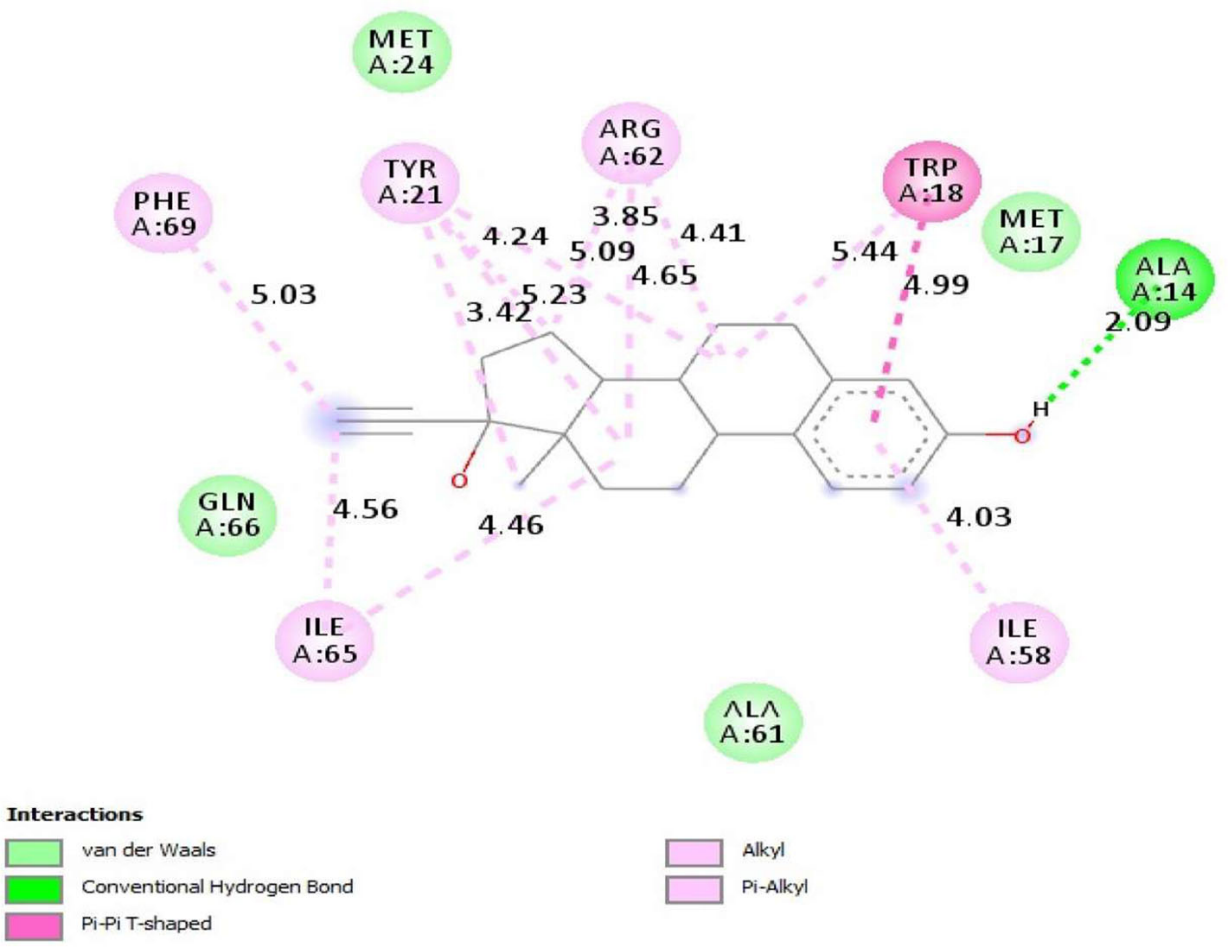
2D Binding of Molecule ETE with 4IP8

**Fig. 9:**
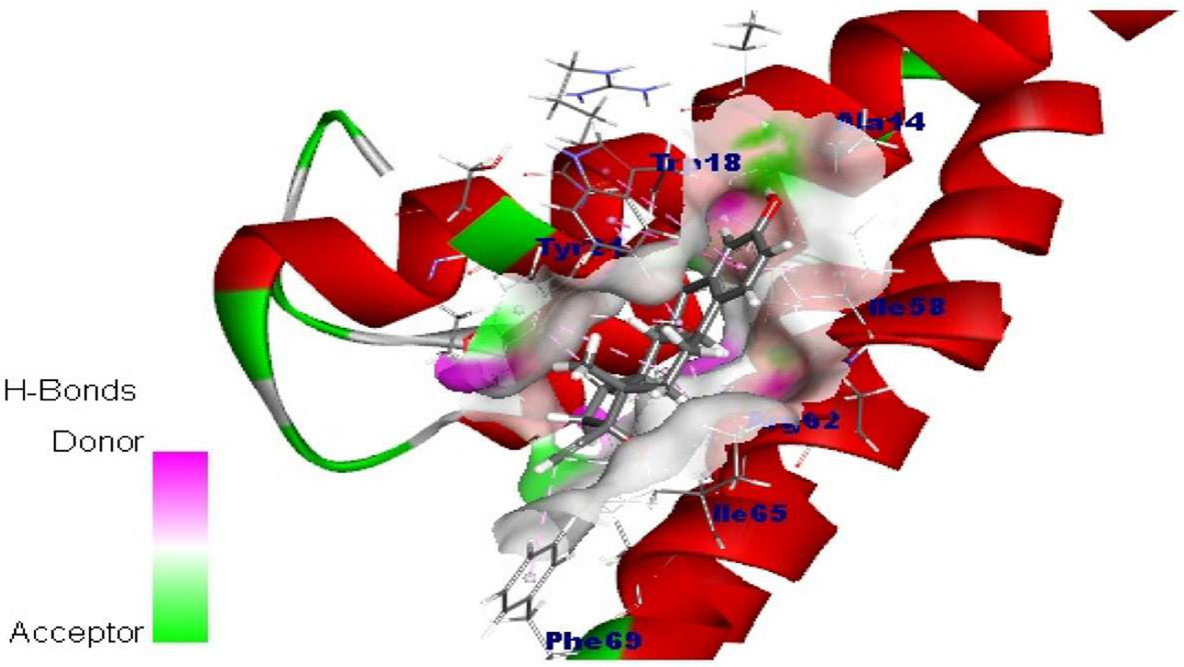
3D Binding of Molecule ETE with 4IP8

In the present analysis, the docking simulations are performed to classify the active site conformation and significant interactions with the receptor binding sites responsible for complex stability. The over expressed genes is recognized in polycystic ovary syndrome and their x-ray crystallographic proteins structure are selected from PDB for docking studies. The standard drugs containing steroid nucleus are most commonly used either alone or in combination with other drugs. The docking studies of standard molecules containing the steroid ring have been carried out using Sybyl X 2.1 drug design software. The docking studies were performed to know the biding interaction of standard molecules on identified overexpressed genes of protein. The X- RAY crystallographic structure of one protein in each of four over expressed genes of POLR2K, RPS15, RPS15 and SAA1 of their co-crystallised protein of PDB code 1LE9, 3OW2, 1G1X and 4IP8 respectively were selected for the docking (Fig. 7). A total of three drug molecules of ethinylestradiol (ETE), levonorgestril (LNG) and desogestril (DSG) were docked with over expressed proteins to assess the binding affinity with proteins. The binding score greater than six are said to be good, all three drug molecules obtained binding score greater than 7 respectively. The molecules ETE obtained with a high binding score of 9.943 with SAA1 of PDB code 4IP8 and 8.260, 8.223 and 8.019 with 1G1X, 3OW2 and 1LE9. The LNG obtained highest binding score of 8.535 with SAA1 of PDB code 4IP8 and 8.351, 7.973 and 7.854 with RPS15, POLR2K and RPS15 alpha of PDB code 3OW2, 1LE9 and 1G1X respectively. DSG: highest with POLR2K of 8.273 with PDB code 1LE9, 8.158 with SAA1 of PDB code 4IP8, 7.745 with RPS15 alpha of PDB code 1G1X and obtained least binding score of 5.674 with RPS15 of PDB code 3OW2 respectively (Table 7). The molecule ETE and LNG has highest binding score its interaction with protein 4IP8 and hydrogen bonding and other bonding interactions with amino acids are depicted by 3D (Fig. 8) and 2D (Fig. 9)

**Table 6:** miRNA - target gene and TF - target gene interaction

Regulation	Target Genes	Degree	MicroRNA	Regulation	Target Genes	Degree	TF
Up	RPL13A	97	hsa-mir-8067	Up	RBX1	15	PER3
Up	RPS15A	95	hsa-mir-4518	Up	RPS15	13	CTCF
Up	RPLP0	71	hsa-mir-3685	Up	RPS20	12	E2F7
Up	ADCY6	65	hsa-mir-1202	Up	ADCY6	11	LMO2
Up	RPS29	48	hsa-mir-4461	Up	POLR2K	9	POLR2H
Up	RPL30	47	hsa-mir-6811-5p	Up	RPS15A	8	FOXF2
Up	RPL35	44	hsa-mir-2278	Up	RPL35	8	MYB
Up	RPS23	40	hsa-mir-4282	Up	RPS23	5	USF1
Up	SAA1	33	hsa-mir-4701-3p	Up	RPL13A	5	NFYA
Up	RPS5	29	hsa-mir-1301-3p	Up	RPS29	5	JUN
Up	RBX1	29	hsa-mir-5187-3p	Up	POLR2J	5	POLR2C
Up	RPS20	26	hsa-mir-708-5p	Up	RPL30	4	IRF7
Up	RPS15	23	hsa-mir-1260b	Up	RPLP0	3	SMAD3
Up	POLR2K	13	hsa-mir-5680	Up	RPS5	3	GABPA
Up	POLR2J	9	hsa-mir-129-2-3p	Up	SAA1	2	CEBPB
Down	CTNND1	129	hsa-mir-8082	Up	PHB2	1	PHB2
Down	ESR1	98	hsa-mir-4422	Down	ESR1	122	NCOA2
Down	NEDD4L	76	hsa-mir-548am-5p	Down	LCK	21	EBF1
Down	KNTC1	62	hsa-mir-6886-3p	Down	GLI3	18	SMAD2
Down	NGFR	56	hsa-mir-9500	Down	NEDD4L	17	JUND
Down	TIAM2	49	hsa-mir-3679-5p	Down	CALCR	15	FOXO3
Down	GLI3	33	hsa-mir-1913	Down	NGFR	10	POU2F1
Down	FASLG	25	hsa-mir-7849-3p	Down	FASLG	8	DAXX
Down	PLD1	22	hsa-mir-3200-3p	Down	CTNND1	7	GATA1
Down	OBSCN	19	hsa-mir-3657	Down	KNTC1	5	SP1
Down	CALCR	12	hsa-mir-4735-5p	Down	PLD1	4	E2F1
Down	PTGDR	11	hsa-mir-4477b	Down	TIAM2	4	FOXD1
Down	CCL28	8	hsa-mir-770-5p	Down	GLI2	1	GLI3
Down	LCK	6	hsa-mir-520c-3p	Down	ERG	1	ESR1
Down	S1PR5	4	hsa-mir-31-5p	Down	PTGDR	1	RORA
				Down	CCL28	1	FOS
				Down	OBSCN	1	CUX1

**Table 7:** Docking results of standard drugs on overexpressed proteins

Sl. No/ Code	Over expressed gene: POLR2K			Over expressed gene: RPS15			Over expressed gene: RPS15 alpha			Over expressed gene: SAA1		
	PDB: 1LE9			PDB: 3OW2			PDB: 1G1X			PDB: 4IP8		
	Total Score	Crash (-Ve)	Polar	Total Score	Crash (-Ve)	Polar	Total Score	Crash (-Ve)	Polar	Total Score	Crash (-Ve)	Polar
ETE	8.019	-0.755	1.219	8.223	-1.768	2.517	8.260	-0.857	3.135	9.943	-1.689	0.891
LNG	7.973	-0.945	1.295	8.351	-2.752	3.465	7.854	-0.599	2.373	8.535	-1.948	0.057
DSG	8.273	-1.124	1.116	5.674	-1.611	2.212	7.745	-1.046	2.306	8.158	-1.997	0.000

## Discussion

PCOS is a most prevalent endocrine disorder with hyperandrogenism and chronic anovulation [29]. If not treated promptly and effectively, PCOS can seriously reduce the quality of life. There is no doubt that considerate syndrome at the molecular level will help to develop their diagnosis and treatment [30]. Up to now, various biomarkers have been identified to be linked with PCOS and might be selected as therapeutic targets, but the detailed mechanism of gene regulation leading to syndrome advancement remains elusive [31].

In our investigation, we aimed to identify biomarkers of PCOS and uncover their biological functions through bioinformatics analysis. Dataset GSE84958 was selected as expression profiling by high throughput sequencing dataset in our analysis. As a result, 360 up regulated and 379 down regulated genes at least 4-fold change between PCOS and normal control samples were screened out. ABI3BP protein expression in heart tissue was significantly related with cardiovascular disease [32], but this gene might be liable for progression of PCOS. Romo-Yáñez et al [33] have revealed the expression of BNIP3 was linked with diabetic in pregnancies, but this gene might be responsible for progression of PCOS. F13A1 is an essential regulatory factor to be associated in PCOS development [34]. An investigation has reported that the ITIH4 can promote non-alcoholic fatty liver disease [35], but this gene might be important for progression of PCOS. Da et al [36] have suggested that the TET3 is an important role in controlling type 2 diabetes progressions, but this gene might be key role in PCOS.

The GO and pathway enrichment analysis was of great importance for interpreting the molecular mechanisms of the key cellular activities in PCOS. RPS5 [37], RBM3 [38], BAK1 [39], NDUFC2 [40], NDUFS4 [41], NDUFS5 [42], UQCRFS1 [43], COX6B1 [44], NDUFA13 [45], PRMT1 [46], RDX (radixin) [47], EPHB4 [48], SYNE2 [49], DNAH5 [50], NEDD4L [51], PDE4B [52] and CTNND1 [53] plays a critical role in the process of cardiovascular disease, but these genes might be linked with development of PCOS. Ostergaard et al [54], Zi et al [55], Kunej et al [56], Van der Schueren et al [57], Jin et al [58], Emdad et al [59], Liu et al [60], Scherag et al [61], Shi and Long [62], Sharma et al [63], Parente et al [64], Saint-Laurent et al [65] and Lee [66] demonstrated that over expression of COA3, PHB (prohibitin), UQCRC1, COX4I1, IFI27, MTDH (metadherin), S100A16, SDCCAG8, GLI2, NTN1, NLGN2, FGFR3 and PTPRN2 could cause obesity, but these genes might be involved in progression of PCOS. Alsters et al [67], Lee et al [68], Shiffman et al [69], Yaghootkar et al [70], Rotroff et al [71], Cheng et al [72], Baig et al [73], Zhang et al [74], Lebailly et al [75], Ferris et al [76], Lempainen et al [77] and McCallum et al [78] presented that high expression of CPE (carboxypeptidase E), RPL13A, CERS2, CCND2, PRPF31, SARM1, PLD1, EPHA4, ARNTL2, BATF3, IKZF4 and MEN1 were associated with diabetes, but these genes might be linked with advancement of PCOS. Wang et al [79], Tian et al [80], Zhang et al [81] and Carr et al [82] demonstrated that over expression of ATP6AP2, FIS1, GRK4 and KCNQ4 were found to be substantially related to hypertension, but these genes might be essential for PCOS progression. Atiomo et al [83], Lara et al [84] and Douma et al [85] were reported that NQO1, NGFR (nerve growth factor receptor) and ESR1 could be an index for PCOS. Jin et al [86] presented that GLI3 was associated with non-alcoholic fatty liver disease, but this gene might be linked with development of PCOS.

In the present investigation, PPI network and its modules has been shown that significant amount of hub gene might be associated with progression of PCOS. Zhang et al [87] proposed that SAA1 was linked with progression of obesity, but this gene might be important for progression of PCOS. Deng et al [88] indicated that ADCY6 was responsible for development of cardiovascular disease, but this gene might be associated with advancement of PCOS. POLR2K, RPS15, RPS15A, ESR1, LCK (LCK proto-oncogene, Src family tyrosine kinase), S1PR5, CCL28, CTNND11, UQCRQ (ubiquinol-cytochrome c reductase complex III subunit VII), UQCRH (ubiquinol-cytochrome c reductase hinge protein), COX7C, COX6C, COX8A, COX5B, COX6A1, COX7A2L, ARHGAP39, OBSCN (obscurin, cytoskeletal calmodulin and titin-interacting RhoGEF) and TIAM2 might be novel biomarkers for PCOS.

MiRNA-target genes and TF-target genes regulatory networks revealed that the miRNAs, TF and target genes were might be involved in PCOS. Hsa-mir-6886-3p was liable for progression of hypertension [89], but this gene might be involved in progression of PCOS. Some investigations determined that expression of PER3 [90] and SMAD2 [91] were associated with diabetes, but these genes might be linked with advancement of PCOS. NCOA2 was found to be associated with advancement of obesity [92], but this gene might be involved in progression of PCOS. Recently, increasing evidence demonstrated that EBF1 was expressed in coronary artery disease [93], but this gene might be responsible for progression of PCOS. FOXO3 was involved in progression of PCOS [94]. RPLP0, RPS29, KNTC1, hsa-mir-8067, hsa-mir-4518, hsa-mir-3685, hsa-mir-1202, hsa-mir-4461, hsa-mir-8082, hsa-mir-4422, hsa-mir-548am-5p, hsa-mir-9500, RBX1, RPS20, CALCR (calcitonin receptor), CTCF (CCCTC-binding factor), E2F7, LMO2, POLR2H and JUND (jun D proto-oncogene) might be novel biomarkers for PCOS.

Among all three of molecules of ethinylestradiol, levonorgestrel and desogetril respectively, ethinylestradiolhas obtained highest binding score (c-score) of 9.943 with protein of PDB code 4IP8 and obtained 8.260, 8.223 and 8.019 with protein of PDB 1G1X, 3OW2 and 1LE9 respectively. The phenolic -OH group in ring A of ethinylestradiol formed favourable bonding interactions with ALA-14 of Chain A and pi-pi bonding interactions of alicyclic ring B TRP-18. Ethinylestradiol also formed alkyl and pi-alkyl interaction of ring B, C and D with TRP-18, ARG-62, TYR-21, PHE-69, ILE-65 and ILE-58. Ethinylestradiol also formed Van der Waals interactions with ACA-61, MET-17, MET-24 and GLN-66 respectively. It is assumed that the highest binding score (c-score) of ethinylestradiol is due to the presence of aromatic ring and the phenolic –OH group.

In conclusion, we used a series of bioinformatics analysis methods to find the crucial genes and pathways associated in PCOS initiation and development from expression profiling by high throughput sequencing containing PCOS samples and normal control samples. Our investigations provide a more specific molecular mechanism for the advancement of PCOS, detail information on the potential biomarkers and therapeutic targets. However, the interacting mechanism and function of genes need to be confirmed in further experiments.

## Data Availability

The datasets supporting the conclusions of this article are available in the GEO (http://www.ncbi.nlm.nih.gov/geo) repository. [(GSE84958) (https://www.ncbi.nlm.nih.gov/geo/query/acc.cgi?acc=GSE84958)]
